# Molecular mechanism on forcible ejection of ATPase inhibitory factor 1 from mitochondrial ATP synthase

**DOI:** 10.1038/s41467-023-37182-9

**Published:** 2023-03-31

**Authors:** Ryohei Kobayashi, Hiroshi Ueno, Kei-ichi Okazaki, Hiroyuki Noji

**Affiliations:** 1 Department of Applied Chemistry, Graduate School of Engineering, The University of Tokyo, Bunkyo-ku, Tokyo 113-8656 Japan; 2 Research Center for Computational Science, Institute for Molecular Science, Okazaki, Aichi 444-8585 Japan; 3 The Graduate University for Advanced Studies, SOKENDAI, Okazaki, Aichi 444-8585 Japan

**Keywords:** Single-molecule biophysics, Kinetics, Enzyme mechanisms, Bioenergetics

## Abstract

IF_1_ is a natural inhibitor protein for mitochondrial F_o_F_1_ ATP synthase that blocks catalysis and rotation of the F_1_ by deeply inserting its N-terminal helices into F_1_. A unique feature of IF_1_ is condition-dependent inhibition; although IF_1_ inhibits ATP hydrolysis by F_1_, IF_1_ inhibition is relieved under ATP synthesis conditions. To elucidate this condition-dependent inhibition mechanism, we have performed single-molecule manipulation experiments on IF_1_-inhibited *bovine* mitochondrial F_1_ (*b*MF_1_). The results show that IF_1_-inhibited F_1_ is efficiently activated only when F_1_ is rotated in the clockwise (ATP synthesis) direction, but not in the counterclockwise direction. The observed rotational-direction-dependent activation explains the condition-dependent mechanism of IF_1_ inhibition. Investigation of mutant IF_1_ with N-terminal truncations shows that the interaction with the γ subunit at the N-terminal regions is crucial for rotational-direction-dependent ejection, and the middle long helix is responsible for the inhibition of F_1_.

## Abstract (web summary)

IF_1_ is a natural inhibitor of mitochondrial F_o_F_1_-ATP synthase, which blocks catalysis and rotation of the F_1_ motor. Here, the authors show the rotational-direction-dependence of activation from IF_1_ inhibition, with IF_1_ being readily dissociated when F_1_ rotates to the clockwise direction.

## Introduction

F_o_F_1_-ATP synthase (F_o_F_1_) is a rotary motor protein that catalyzes ATP synthesis reaction from ADP and inorganic phosphate (P_i_) using the proton motive force (*pmf*) across membranes. F_o_F_1_ is composed of two rotary motor proteins: F_o_ and F_1_^1–5^. F_o_ is a membrane-embedded protein complex forming the proton translocation pathway. When F_o_F_1_ synthesizes ATP, F_o_ transports protons from the outside to the inside of the membrane, accompanied by the clockwise rotation of the rotor complex inside F_o_F_1_ when viewed from the outside of the membrane. F_1_ is a water-soluble portion of F_o_F_1_ that contains catalytic centers for ATP synthesis. Proton translocation by F_o_ and ATP synthesis/hydrolysis reactions in F_1_ are tightly coupled through the rotation of the rotor complex. When *pmf* is sufficiently high, F_o_ forcibly rotates F_1_, powered by proton translocation down *pmf*, resulting in ATP synthesis via F_1_. When *pmf* is insufficient, F_1_ reverses the rotation and hydrolyzes ATP, which induces F_o_ to pump protons to generate *pmf* ^6^.

**Fig. 1:**
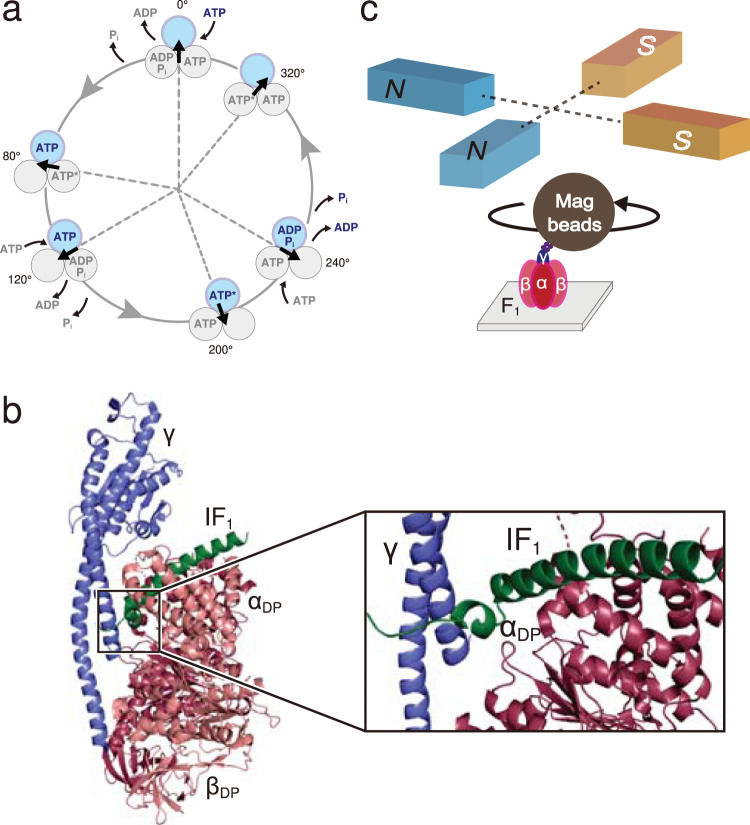
Outline of this work. **a** Rotation scheme of *b*MF_1_. The circles represent the catalytic states of bound nucleotides at the β subunit. ATP* in the circles at 80°, 200°, and 320° represents the catalytically active state in which the bound ATP is to be hydrolyzed. The arrows represent the rotary angles of γ subunit. 0° is defined as the position of the γ subunit where the blue β subunit binds to ATP. *Short pauses* at 10°-20°, 130°-140°, and 250°-260°, observed in our previous paper^20^, are omitted from this figure for clarity. **b** Crystal structure of *b*MF_1_ with IF_1_ bound to the αβ_DP_ subunit (PDB: 2v7q). α_DP_, β_DP_, γ, and IF_1_ are colored by dark red, pink, blue, and green, respectively. The β_DP_ subunit has been omitted for clarity in the enlarged figure. **c** An illustration of the single-molecule rotation assay system of *b*MF_1_. The stator α_3_β_3_-ring is immobilized on a glass surface. A magnetic bead (φ ~ 300 nm) is attached to the rotor γ subunit as a rotation probe via biotin-streptavidin interaction. Magnetic tweezers, consisting of two sets of electromagnets, were equipped onto the sample stage of the microscope.

F_1_ is an ATPase that hydrolyzes ATP to rotate its rotor part counterclockwise, when viewed from the membrane side, against the surrounding stator α_3_β_3_-ring^7^, where the central rotor γ subunit is accommodated in the central cavity^8–12^. F_1_ possesses three catalytic sites for ATP hydrolysis/synthesis, each at the interface between the α and β subunits. Amino acid residues critical for catalysis are mainly located in the β subunit. The “ground-state” crystal structure of *b*MF_1_^13^ revealed that the three β subunits have different conformations and nucleotide-bound states; β with nucleotide analog (β_TP_), β with ADP (β_DP_), and β with none (β_E_). The β_TP_ and β_DP_ adopt a closed form with their C-terminal domain swinging towards the γ subunit, whereas β_E_ adopts an open conformation^4,12,14^. The rotary dynamics of F_1_ have been investigated extensively using single-molecule studies^1,15–19^. The reaction scheme of rotary catalysis in *b*MF_1_^20^ (Fig. 1a) was proposed by considering the structural features of *b*MF_1_ and kinetic analysis in single-molecule studies on F_1_ from thermophilic *Bacillus* PS3 (TF_1_)^21^. Several experiments have shown that most of the crystal structures of *b*MF_1_ represent catalytic dwell states^22–24^.

Most F_o_F_1_ play the primary role in ATP synthesis in vivo, although proton- or sodium-pumping activity driven by ATP hydrolysis is dominant in some species. In general, ATP hydrolysis activity of F_1_ can result in futile ATP consumption. Therefore, several types of regulatory systems can suppress or block the ATP hydrolysis activity of F_1_^25–30^. The most common mechanism universally found in bacterial and mammalian F_1_ is ADP inhibition in which F_1_ spontaneously lapses into a resting state during rotation^31–33^. Mitochondrial F_1_s have a unique inhibitor protein, termed the ATPase inhibitory factor 1 (IF_1_)^34^. It is found in eukaryotic cells and is highly conserved particularly among mammalian cells^25^. Unlike the ε subunit^9,35–38^ that some of the bacterial F_1_s employ for inhibition, IF_1_ is not a built-in inhibitor, but a dissociative one that associates with F_1_ when pH decreases to acidic state or F_1_ is isolated before association with F_o_ to form the whole complex of F_o_F_1_^6^. The most remarkable feature of IF_1_ is its condition-dependent manner of inhibition: IF_1_ inhibits ATP hydrolysis activity of F_1_ almost completely under ATP hydrolysis conditions, whereas, in ATP synthesis conditions, IF_1_ dissociates from F_1_ and does not interfere with ATP synthesis reactions after dissociation^34,39^. Notably, a few recent studies claim that IF_1_ also suppresses the rate of ATP synthesis when IF_1_ is overexpressed in cells^40,41^.

The atomic details of the interaction between IF_1_ and *b*MF_1_ have been investigated in structural studies^42–44^. Full-length bovine mitochondrial IF_1_ forms a homodimer complex via an antiparallel coiled coil^45^, which associates two molecules of the F_1_-ATPase with each N-terminal region, whereas the C-terminal region of IF_1_ forms a coiled-coil structure for dimerization^42^. Deletion of the C-terminal residues 61–84 produces a stable monomeric form of IF_1_, which achieves full inhibition capacity irrespective of pH change^46^. This simple platform of IF_1_ is often used in biochemical^47,48^ and structural studies^43,44^, including this study. The crystal structure of the *b*MF_1_-IF_1_ complex^44^ showed that the long α-helical structure in the middle section of IF_1_ was bound to the interface of the αβ_DP_ pair, whereas the short helix of the N-terminus was in contact with the γ subunit. The N-terminal short helix was linked to the middle-long helix via a distinct kink (Fig. 1b). The crystal structure of *b*MF_1_ with three IF_1_ units, referred to as *b*MF_1_-(IF_1_)_3_, has been resolved^43^, where each αβ interface was bound with IF_1_. Because the structures of the αβ interface are different, IF_1_’s show different conformational states. The structure of IF_1_ on αβ_DP_ is consistent with that of the 1:1 *b*MF_1_-IF_1_ complex, whereas the N-terminal short helix of IF_1_ is not resolved on αβ_TP_. The structure of IF_1_ on αβ_E_ shows the second half of a long helix, and the remaining was unsolved. Based on these observations, the progressive folding of IF_1_ coupled with γ rotation has been proposed. Progressive folding process has also been suggested in biochemical studies^48,49^, although the progressive processes are simplified as a two-step process: the initial binding process and the subsequent isomerization process.

Compared to studies under hydrolysis conditions^48,50,51^, studies on IF_1_ under synthetic conditions are less advanced, despite its importance for the understanding of the condition-dependent inhibition mechanism of IF_1_. IF_1_-inhibited state of F_1_-ATPase is known to be so stable that F_1_ is unable to eject IF_1_ by thermal agitation alone^48^. A typical condition for the unlocking from IF_1_ inhibition is to charge sufficiently high *pmf* to membrane vesicles containing IF_1_-inhibited F_o_F_1_^52–57^. However, the principal mechanism for IF_1_ ejection from the catalytic site of F_1_, followed by the recovery of catalysis in F_1_ (hereafter, “activation from IF_1_ inhibition” in this paper), remains elusive. Many fundamental questions are unsolved such as ‘Does reversible rotation lead to activation from IF_1_ inhibition?’, ‘Are there factors to enhance the activation?’, and ‘Which interactions among IF_1_ and F_1_ are responsible for IF_1_ inhibition and for condition-dependent inhibition?’.

This study investigates the experimental conditions required for the dissociation of IF_1_ from the inhibition complex using magnetic tweezers, which enable control of the angular orientation of the rotor during single-molecule rotation assays for *b*MF_1_ (Fig. 1c). Similar to the mechanical activation of F_1_ from the ADP-inhibited form, we forcibly rotate the rotor of IF_1_-inhibited F_1_, and define activation as the resumption of F_1_ molecule rotation. The activation probability is determined as a function of the angle, similar to our previous stall-and-release experiments^20,21,33,58^. Further, we investigate the roles of the N-terminal short helix and the middle-long helix in inhibition and activation. These results highlight the molecular mechanism of IF_1_ dissociation, which is critical for the unidirectional inhibition mechanism of IF_1_.

## Results

### IF_1_-inhibited states of *b*MF_1_

The rotation of *b*MF_1_ was monitored using magnetic beads (beads diameter, φ ~300 nm) attached to the γ subunit as a rotation probe and recorded at 30 frames per second (fps). During rotation, *b*MF_1_ showed a transient pause in the absence of IF_1_^20^. This was attributed to ADP inhibition. Supplementary Fig. 1 summarizes the kinetic analyses of ADP inhibition. Although it is dependent on ATP concentrations, the mean time for ADP inhibition range between 10–30 s. For IF_1_ inhibition, monomeric bovine IF_1_ (Δ61–84) was used, as described in previous studies^47,48^.

**Fig. 2:**
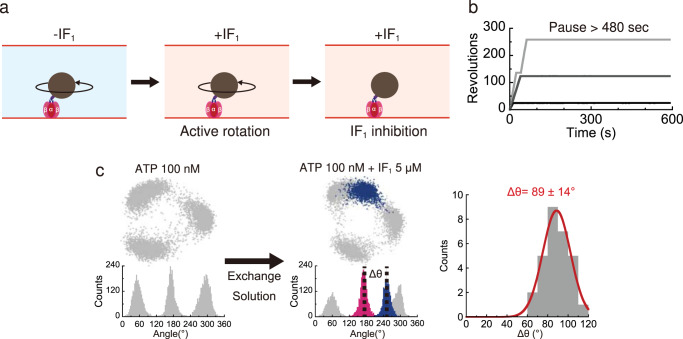
Single-molecule analysis of IF_1_-inhibited *b*MF_1_. **a** Experimental method. After observing a rotating *b*MF_1_ molecule in IF_1_-free solution, we introduced a mixed solution with IF_1_ into the reaction chamber. The *b*MF_1_ continued to rotate for a while but finally stopped rotation. **b** Typical time courses of *b*MF_1_ in the presence of 3 μM IF_1_ and 1 mM ATP. See Supplementary Fig. 2 for more detailed analyses. Source data are provided in the Source Data file. **c** Stall positions of IF_1_ inhibition. (*Left*) An example of the IF_1_-inhibited pauses. After observing the ATP-binding waiting dwell at 100 nM ATP, 5 μM IF_1_ with 100 nM ATP was introduced into the reaction chamber. Blue data points represent stalls of IF_1_ inhibition. (*Right*) The angular distance (Δθ) of IF_1_-inhibited states from the left-side ATP-binding waiting dwell (pink) (*N* = 29 pauses). Values represent mean ± SD estimated from Gaussian fitting of the plots. Source data are provided in the Source Data file.

The experimental procedure for the analysis and manipulation of IF_1_-inhibited *b*MF_1_ molecules was as follows (Fig. 2a); after identification of rotating particles in the presence of 1 mM Mg-ATP, a solution containing IF_1_ and Mg-ATP was gently introduced into the flow cell. After rotations for several tens of seconds, all *b*MF_1_ molecules stopped rotation without exception (Fig. 2b), and none of the observed molecules resumed rotation during the observation time of 480 s (Supplementary Fig. 2a). The pause duration was evidently longer than the duration of ADP inhibition, and thus attributed to IF_1_ inhibition. Under 3 μM IF_1_ and 1 mM ATP, the mean time to lapse into IF_1_ inhibition was approximately 20 s (Supplementary Fig. 2b). The time constant for IF_1_ inhibition obtained in our biochemical experiments at saturated ATP and IF_1_ concentrations (Supplementary Fig. 9, gray) was 30 s, which is approximately 1.5 times longer than that in the single-molecule rotation assay. This is probably due to differences in experimental conditions; the single-molecule rotation assay selectively analyzes actively rotating molecules, whereas biochemical measurement is based on ensemble averaging of active and ADP-inhibited molecules. The latter would require a longer time to lapse into IF_1_ inhibition than actively rotating molecules when ADP-inhibited form is off the pathway to IF_1_ inhibition.

Next, we investigated the dwell position of *b*MF_1_ when inhibited by IF_1_. Experiments were performed under 100 nM ATP condition, where *b*MF_1_ showed three distinct pauses at the ATP-binding dwell angles (Fig. 2c). Transient pauses, such as ADP inhibition, are rarely observed^20^. When a solution containing 100 nM ATP and 5 μM IF_1_ was introduced into the flow chamber, *b*MF_1_ stopped rotating completely. The time constant for IF_1_ inhibition at 100 nM ATP was 461 s (Supplementary Fig. 3), which was longer than that obtained with 1 mM ATP, i.e., 19.6 s (Supplementary Fig. 2b). The observed ATP-dependent effect of IF_1_ inhibition was consistent with previous biochemical analysis^48,49,59,60^. Fig 2c shows a representative dataset for the angular position analysis of IF_1_ inhibition. The angular position of IF_1_ inhibition (Fig. 2c, blue) was determined using the ATP-binding dwell angles as the reference; it was defined as the angular distance from the left-side ATP-binding pause (Fig. 2c, pink). The position of IF_1_ inhibition was determined as 89 ± 14° from the ATP-binding angle. This value was almost identical to the pause positions of *b*MF_1_ stalled by AMP-PNP (76°)^20^ and sodium azide (79° in Supplementary Fig. 4), which corresponds to the position of the catalytic dwell (80°). Thus, we confirmed that IF_1_-inhibited *b*MF_1_ pauses at the catalytic dwell angle, as seen in human mitochondrial F_1_^19^.

### Activation of IF_1_-inhibited *b*MF_1_ via forcible rotation

**Fig. 3:**
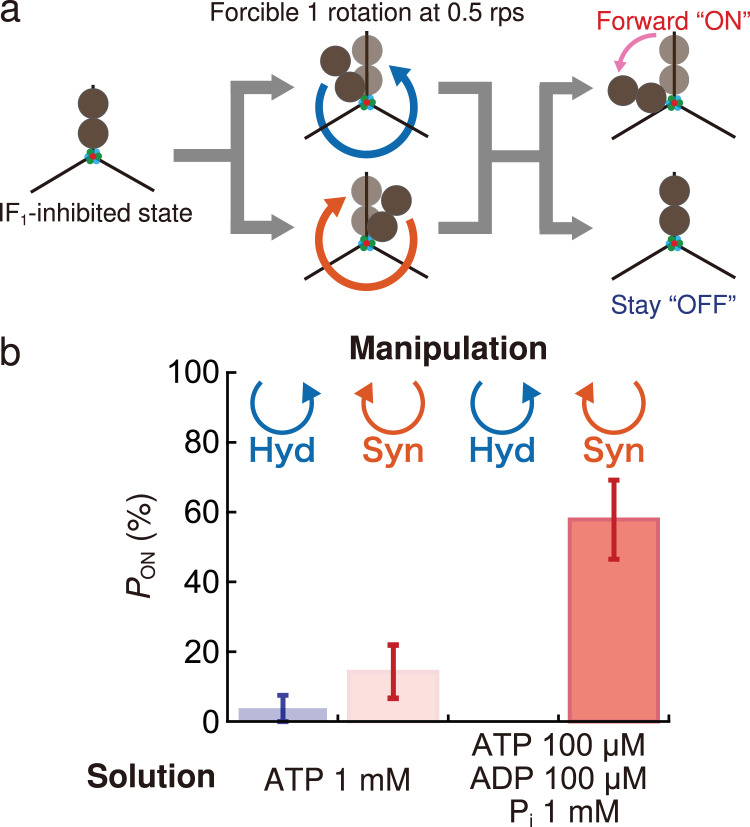
Single-molecule manipulation of IF_1_-inhibited *b*MF_1_. **a** Schematic images of the manipulation procedure. When *b*MF_1_ was stalled by IF_1_, the magnetic tweezers were turned on to stall *b*MF_1_ to rotate one clockwise or counterclockwise revolution at the rate of 0.5 revolutions per second (rps). After manipulation, released *b*MF_1_ either resumed its rotation (ON) or did not (OFF). These behaviors indicate whether or not IF_1_ dissociates from *b*MF_1_ under the stalling time. **b** Reactivation probability of IF_1_-inhibited *b*MF_1_ after manipulation. “Hyd” and “Syn” represent the direction of hydrolysis (counterclockwise) and synthesis (clockwise), respectively. Values represent reactivation probability (${P}_{{ON}}$) ± SD. ${P}_{{ON}}$ was defined as the probability of an ON event against total molecules. The SD of ${P}_{{ON}}$ is given as $\sqrt{{P}_{{ON}}\,(100-{P}_{{ON}})/N}$, where $N$ is the number of total molecules (*N* = 19-26 molecules). Source data and the exact number of molecules in each data point are provided as the Source Data file.

In the rotation assays, IF_1_-inhibited *b*MF_1_ did not resume the rotations once it lapsed into IF_1_ inhibition. By contrast, it was reported that F_o_F_1_ is activated from IF_1_ inhibition when the *pmf* is charged on the vesicle membrane, in which the F_o_F_1_ is embedded^55^. To explore the crucial conditions and factors for unlocking from IF_1_ inhibition, IF_1_-inhibited *b*MF_1_ molecules were forcibly rotated using magnetic tweezers. First, we tested the clockwise rotation for one turn (Fig. 3a), considering that forcible rotation in the ATP synthesis direction for one turn is sufficient for the unlock from IF_1_ inhibition. Before the forcible rotation, the buffer in a flow cell was exchanged with IF_1_-free ATP solution (1 mM) to prevent possible rebinding of IF_1_. However, the molecules did not show activation in most cases (Fig. 3b). Only a small fraction of molecules (10%) resumed continuous rotation. When forcibly rotated in the counterclockwise direction, the activation probability was even lower (<4%), suggesting the rotational-direction-dependence for the activation from the IF_1_ inhibition. The reactivation is a unique phenomenon that was never observed without manipulation with magnetic tweezers, but the probability is too low compared to the reported *pmf-*induced full activation of F_o_F_1_^52–57^.

Next, we tested the effect of ADP and P_i_ in a forcible rotation to further mimic ATP synthesis conditions. After confirming IF_1_ inhibition, the buffer in the flow cell was gently exchanged with IF_1_-free ATP synthesis buffer (100 μM ATP, 100 μM ADP, 1 mM P_i_), and then, IF_1_-inhibited *b*MF_1_ molecules were forcibly rotated in the clockwise or counterclockwise direction for 360°. As shown in Fig. 3b, a remarkable increase in activation was observed when IF_1_-inhibited *b*MF_1_ was rotated in the clockwise direction in the presence of ADP and P_i_; the probability of activation was about 60%. This is in contrast to the reactivation probability for the clockwise manipulation in ADP/P_i_-free solution (10%). An evident rotational-direction-dependent manner was observed; the counterclockwise rotation failed the activation. These observations indicate that both directional manipulation and the presence of substrates for ATP synthesis are requisite for the efficient activation of the IF_1_-inhibited state. To investigate the effect of individual substrates on activation, we performed the same manipulation experiment in the presence of ADP or P_i_, respectively (Supplementary Fig. 5). P_i_ was more effective for reactivation (28%), whereas the activation achieved only 4% in the presence of ADP. Notably, once activated, *b*MF_1_ molecules did not show long pauses that are attributable to IF_1_ inhibition in IF_1_-free solution. This observation indicates that activation is accompanied by dissociation of IF_1_ from F_1_.

### Angle-dependence of IF_1_ dissociation

**Fig. 4:**
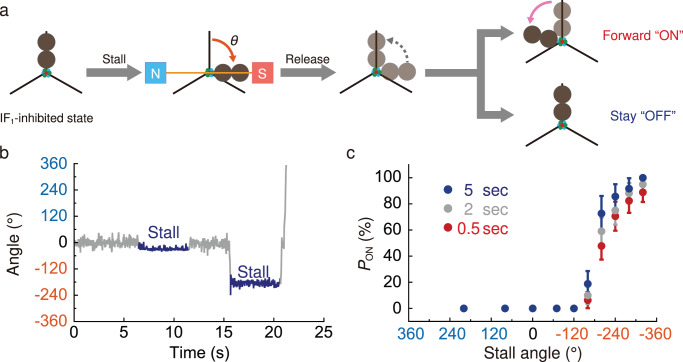
The stall-and-release experiment of IF_1_-stalled *b*MF_1_. **a** Schematic images of the manipulation procedure in the stall-and-release experiment. When *b*MF_1_ was stalled by IF_1_, the magnetic tweezers were turned on to stall *b*MF_1_ at the target angle. After the set time had elapsed, the magnetic tweezers were turned off, and the molecule returned to the initial angle. Released *b*MF_1_ either resumes its rotation (ON) or stays at the initial position (OFF). These behaviors indicate whether or not IF_1_ dissociates from *b*MF_1_ under the stalling time. **b** A representative time course of the stall-and-release experiment under 100 μM ATP, 100 μM ADP, and 1 mM P_i_ in the presence of 3 μM IF_1_. In this figure, the stall time for both trials (blue) was 5 s and the stall angles were -26° and -168°, respectively. **c** Angle dependence of reactivation probability under 100 μM ATP, 100 μM ADP, and 1 mM P_i_ in the presence of 3 μM IF_1_. Each data point was obtained from 10 to 57 trials using 4 to 14 molecules. Counterclockwise rotation (blue) and clockwise rotation (orange) are defined as positive and negative direction, respectively. The colors on the plots represent the stall time of 0.5 s (red), 2 s (gray) and 5 s (blue), respectively. Values represent reactivation probability (${P}_{{ON}}$) ± SD. ${P}_{{ON}}$ was defined as the probability of an ON event against total trials. The SD of ${P}_{{ON}}$ is given as $\sqrt{{P}_{{ON}}\,(100-{P}_{{ON}})/N}$, where $N$ is the number of total trials in each data point. Source data and the exact number of trials in each data point are provided in the Source Data file.

We determined the activation probability as a function of the rotation angle by performing a “stall-and-release” experiment. The experimental procedure was as follows (Fig. 4a); after confirming IF_1_ inhibition, the inhibited *b*MF_1_ molecules were rotated to stall at the target angle for the programmed period of 0.5–5.0 s with the magnetic tweezers. After the set time had elapsed, the magnetic tweezers were turned off to release the F_1_ molecule. The released molecule showed two types of behaviors (Fig. 4b): starting active rotation or returning to the initial position of IF_1_ inhibition to resume the pause. The former behavior was identified as activation from IF_1_ inhibition, and the latter as failure of activation. In contrast to the abovementioned activation by a forcible 360° rotation, we conducted a stall-and-release experiment without washing IF_1_ in solution. The molecules again lapsed into IF_1_ inhibition after activation due to the rebinding of IF_1_ in a solution containing 3 μM IF_1_. The mean rotation time until the re-inactivation of activated F_1_ molecules was 18.3 s (Supplementary Fig. 6), and the result is consistent with the time constant of IF_1_ inhibition (15.2 s), indicating that re-inactivation was due to the rebinding of IF_1_ from solution. We repeated the manipulation of each molecule to confirm reproducibility. After manipulation, a few molecules occasionally exhibited unusual behaviors, such as random tethered Brownian motion, and nonspecific binding to or detachment from the coverslip. These data were omitted from the analyses. A detailed description of the analysis of molecules in this experiment is presented in Supplementary Note and Supplementary Fig. 7.

Experiments were conducted with 100 μM ATP, 100 μM ADP, and 1 mM P_i_. Fig 4c shows the experimental results, where the counterclockwise direction of the stall angle was defined as positive relative to the initial IF_1_-inhibited state. In principle, activation was rarely observed in the counterclockwise manipulation, which is consistent with the experimental results for forcible 360° rotation manipulation (Fig. 3b). For the clockwise manipulation, rotation up to 120° did not activate IF_1_-inhibited *b*MF_1_. When rotated over 200°, the activation probability, i.e., *P*_ON_ was remarkably increased. *P*_ON_ was 60% when stalled at −200° for 2 s, and reached over 90% when stalled at –320° for 5 s. At each stall angle, *P*_ON_ increased with stall times. These features are similar to those observed in our previous studies on the angle dependence of ATP binding and hydrolysis in TF_1_^58^ and *b*MF_1_^20^ as well as the angle dependence of activation from ADP inhibition^32^. These results on angle-dependent activation are discussed in detail in the *Discussion* section.

### N-terminal-truncated mutants of IF_1_

**Fig. 5:**
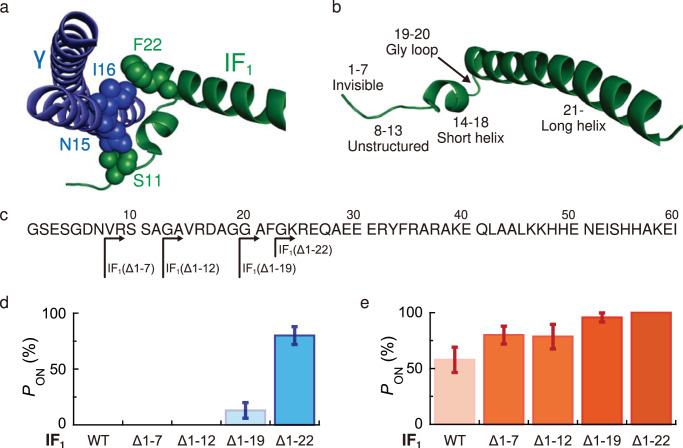
Analysis of mutant IF_1_’s with the N-terminal truncation. **a** Enlarged illustration of the interactions between IF_1_ and γ subunit in the *b*MF_1_-IF_1_ complex (PDB: 2v7q). S11 and F22 in IF_1_ (green) can interact with N15 and I16 in γ subunit (blue), respectively. **b** Details of the IF_1_ structure (PDB: 2v7q), where residues 8–50 are resolved. Residues 1–7 are not resolved. Residues 8-13 are resolved and form an extended structure. Residues 14–18 and 21–50 form short and long helix, respectively, linked by glycine loop in residues 19–20. **c** Sequence of bovine IF_1_^1–60^ and definition of N-terminal truncated mutants. **d**, **e** Reactivation probability of inhibited *b*MF_1_ by mutant IF_1_s after **d** counterclockwise and **e** clockwise manipulation. Experiments were performed under 100 μM ATP, 100 μM ADP, and 1 mM P_i_. The data for IF_1_(WT) are also plotted for comparison (Fig. 3b). Values represent reactivation probability (${P}_{{ON}}$) ± SD. ${P}_{{ON}}$ was defined as the probability of an ON event against total molecules. The SD of ${P}_{{ON}}$ is given as $\sqrt{{P}_{{ON}}\,(100-{P}_{{ON}})/N}$, where $N$ is the number of total molecules (*N* = 11–25 molecules). Source data and the exact number of molecules in each data point are provided in the Source Data file.

With an aim to investigate which structural elements of IF_1_ are responsible for the observed rotation-direction-dependent activation, we generated IF_1_ mutants with N-terminal truncations and tested their inhibitory functions in biochemical and single-molecule manipulation experiments (Fig. 5). IF_1_ consists of the N-terminal region that is missing in the crystal structure (1–7), unstructured region (8–13), the short helix region (14–18), glycine loop (19–20), and the long helix region (21–60) (Fig. 5a, b). The long helix of IF_1_ mainly interacts with the β_DP_ subunit, whereas the proximity contacts with the γ subunit are found at S11 and F22 (Fig. 5a)^43^. We prepared four truncation mutants of IF_1_: IF_1_(Δ1–7), IF_1_(Δ1–12), IF_1_(Δ1–19), and IF_1_(Δ1–22) (Fig. 5c). The inhibitory effects of the mutants were first analyzed using a solution ATPase assay (Supplementary Figs. 8 and 9). IF_1_(Δ1–7) and IF_1_(Δ1–12) showed similar inhibitory effects to IF_1_(WT); upon association with *b*MF_1_, the mutants of IF_1_ completely inhibited ATPase activity. In contrast, IF_1_(Δ1–19) and IF_1_(Δ1–22) showed a reduced inhibitory effect. At concentrations lower than 0.5 μM, IF_1_(Δ1–19) did not completely inhibit ATPase activity, suggesting a reversible association/dissociation. IF_1_(Δ1–22) showed a lower inhibitory effect with a peculiar kinetic behavior. Upon addition of IF_1_(Δ1–22) into the assay mixture, ATPase activity decreased, followed by slow recovery. Although the mechanisms underlying such complex behavior are unclear, these biochemical measurements showed that the inhibitory effect of IF_1_(Δ1–19) and IF_1_(Δ1–22) was less than that of IF_1_(Δ1–7) and IF_1_(Δ1–12).

We analyzed the rotation of *b*MF_1_ in the presence of truncated mutants of IF_1_. Similar to the biochemical experiments, we observed that IF_1_(Δ1–7) and IF_1_(Δ1–12) showed nearly the same properties as those of IF_1_(WT) (Supplementary Fig. 10). As shown in the time course and pause time analysis, inhibition by these mutants was essentially irreversible; IF_1_-inhibited *b*MF_1_ did not spontaneously resume active rotations within the observation time. In contrast, further N-terminal truncation had a significant effect on IF_1_ inhibition. With IF_1_(Δ1–19) and IF_1_(Δ1–22), *b*MF_1_ molecules did not completely stop the rotation, but the molecules showed frequent transitions between rotation and intervening pauses (Supplementary Fig. 10j and m). The mean duration of pauses with IF_1_(Δ1–19) or IF_1_(Δ1–22) was 250 s or 60 s, respectively. These observations show that the short helix and N-terminus of the long helix of IF_1_ have critical roles in the stabilization of IF_1_ inhibition.

Rotation-direction-dependent activation was investigated with the mutant IF_1_’s. Whereas the activation experiments for IF_1_(Δ1–7) and IF_1_(Δ1–12) were conducted in IF_1_-free solutions, those were done in the presence of IF_1_ for IF_1_(Δ1–19) and IF_1_(Δ1–22). This is because these mutants can dissociate from F_1_ during buffer exchange, hampering the activation experiment. Fig 5d and e show the activation probabilities after a forcible 360° rotation in the counterclockwise and clockwise directions, respectively. The probabilities of activation from inhibition with IF_1_(Δ1–7) and IF_1_(Δ1–12) showed similar trends to that of IF_1_(WT) in both directions; i.e., no activation after counterclockwise rotation, whereas clockwise rotation induced activation with a significant probability of 80%, which is higher than that for IF_1_(WT) at 58%. These results indicate that the unstructured N-terminal region from positions 1 to 13 of IF_1_ is not responsible for the principal mechanism of IF_1_ inhibition, although the region contributes to the structural stabilization of the *b*MF_1_-IF_1_ complex. Unlike the truncation of the unstructured N-terminal region, the truncation of the short helix and the subsequent N-terminal region of the long helix had the distinctive impact on rotation-direction-dependent activation. For the mutants IF_1_(Δ1–19) and IF_1_(Δ1–22), the activation probabilities after clockwise or counterclockwise rotation were both higher than those for IF_1_(WT), IF_1_(Δ1–7), and IF_1_(Δ1–12). In particular, a significant fraction of events showed activation after counterclockwise rotation: 13% for IF_1_(Δ1–19) and 80% for IF_1_(Δ1–22). The activation probability after clockwise rotation was also higher than that for IF_1_(WT), IF_1_(Δ1–7), and IF_1_(Δ1–12), and was close to 100%. These results suggest that IF_1_ readily dissociates from *b*MF_1_ without the short helix and the N-terminal tip of the long helix, as suggested by biochemical data. Importantly, IF_1_(Δ1–22) almost loses the rotation-direction-dependent feature, and the activation probabilities after clockwise or counterclockwise rotation are more than 80%. Considering that IF_1_ shows direct contact with the γ subunit in the truncated regions, these results suggest that the rotation-direction-dependent dissociation mechanism is based on contact with the γ subunit. One may consider that IF_1_(Δ1–22) dissociates from *b*MF_1_ not by forcible rotation, but by its nature of spontaneous dissociation. However, the manipulation time is 2 s which is too short for spontaneous dissociation of IF_1_(Δ1-22); the probability of spontaneous dissociation within 2 s is only 3% when estimated from the mean time constant of spontaneous activation, 60 s (see Supplementary Note). Thus, the observed activation with IF_1_(Δ1–22) results due to forcible rotation.

## Discussion

In this study, we observed the activation from IF_1_ inhibition by forcible rotation with magnetic tweezers. Activation is accompanied by the ejection of bound IF_1_ from *b*MF_1_. This is supported by the following observations: re-inactivation by IF_1_ was observed only when the solution contained IF_1_. In the absence of IF_1_, the activated molecules did not show long pauses attributable to IF_1_ inhibition. Second, the mean rotation time before re-inactivation was in good agreement with that for IF_1_ inhibition (Supplementary Fig. 6). The analysis of activation from IF_1_ inhibition provides important implications for the mechanism of IF_1_ inhibition. *b*MF_1_ molecules during IF_1_ inhibition were activated when the γ subunit was forcibly rotated with magnetic tweezers for over 200° in the clockwise (synthesis) direction. The activation probability was significantly enhanced in the presence of P_i_ in the solution by a factor of 15, although ADP also had an impact on the activation by a factor of 2 or more (Supplementary Fig. 5). Thus, it is evident that reactivation from IF_1_ inhibition occurs under ATP synthesis conditions. With IF_1_(WT), the probability of activation reached 100% when stalled at –320° for 5 s. This is sufficiently high to explain the *pmf*-induced activation of the IF_1_-inhibited F_o_F_1_^52–57^. Thus, the principal mechanism for the *pmf*-induced activation of IF_1_-inhibited F_o_F_1_ is the ejection of IF_1_ from F_1_ by forcible rotation powered by *pmf*-driven F_o_ motor in the F_o_F_1_ complex. The requirement of ADP and P_i_ for efficient activation indicates that forcible clockwise rotation should be coupled with the ATP synthesis reaction for IF_1_ ejection. As the presence of substrates enhances the cooperative nature of F_1_, IF_1_ may likely be ejected through a concerted conformational transition of the α and β subunits coupled with γ subunit rotation.

**Fig. 6:**
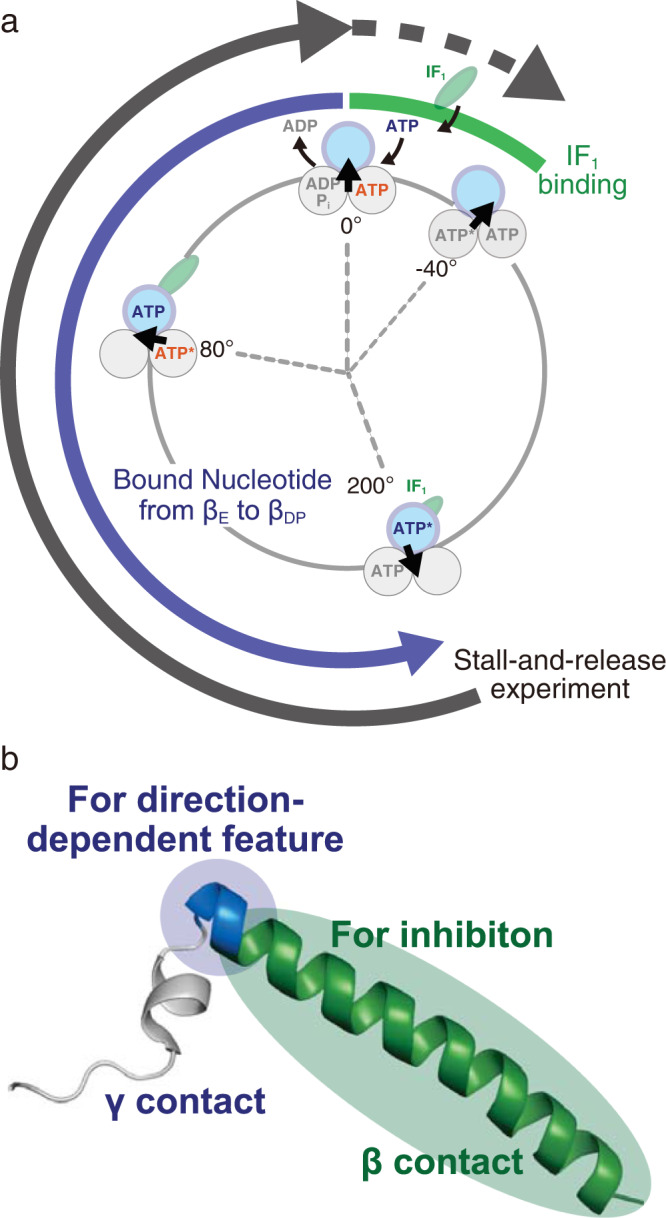
The proposed mechanisms presented in this study. **a** Coupling scheme of IF_1_ dissociation with the rotary mechanism in *b*MF_1_. A part of the reaction scheme of rotary catalysis in *b*MF_1_ (Fig. 1a) is described with only the essential elements. The green oval represents IF_1_. The green line in the scheme represents the IF_1_ binding angle in the range of –40°-0°. The blue line represents the angular difference (0°–200°) required for the conformational transition of the blue β subunit from β_E_ to β_DP_ via β_TP_. The black line represents the angle distance for IF_1_ dissociation, estimated in the stall-and-release experiment. **b** Schematic representation of the role of IF_1_ (PDB: 2v7q). The N-terminal region of IF_1_ wraps around the γ subunit, and the long helix of IF_1_ interacts with the β_DP_ subunit. In addition to the N-terminal amino acid residues, including the short helix (residues 14-18), residues 20–22 (blue) play a critical role in the rotation-direction-dependent activation. Residues starting at residue 23 (green) in the central long helix work as a prototypical inhibitor.

We observed a significant increase in the probability of activation when the IF_1_-inhibited *b*MF_1_ was rotated over –200° (Fig. 4c). The crystal structure of *b*MF_1_-IF_1_ complex show that IF_1_ binds to β_DP_ which represents the +200° state from the ATP-binding state (0°) in the scheme. These results suggest that IF_1_ is ejected through the state transitions of the β subunit from the +200° state to the 0° state or –40° (Fig. 6a). This state transition should be coupled with the conformational transition of the β subunit from a closed to an open conformation. The closed-to-open transition accompanies the swing-out motion of the C-terminal domain of the β subunit to which IF_1_ binds via the long α helix. Thus, IF_1_ dissociates from the β subunit in an open conformation that facilitates IF_1_ dissociation. The closed-to-open conformational transition would destabilize the *b*MF_1_-IF_1_ complex by pulling the N-terminal regions of IF_1_ out of the γ subunit, because the C-terminal domain of the β subunit in the open conformation is apart from the axis of the γ subunit, compared with the closed conformation. This dissociation model is almost the reverse process of the IF_1_ association proposed for the *b*MF_1_-(IF_1_)_3_ structure, which suggests the progressive folding of IF_1_ coupled with the conformational transition of the β subunit from β_E_ to β_DP_ via β_TP_.

Estimating the energy required for activation from IF_1_ inhibition is important to discuss under what conditions ATP synthase is activated in the cell. Although this study does not measure the torque of magnetic tweezers applied to F_1_ molecules, rough estimation is possible based on several assumptions. When *b*MF_1_ generates torque of 40 pN·nm like other F_1_s, the minimum value for proton motive force as voltage to reverse F_1_ is 196 mV, considering the proton stoichiometry of 8 protons/turn. In addition, the minimum energy for IF_1_ ejection is assumed to be 140 pN·nm (84 kJ/mol).

The above discussion indicates the reversible association/dissociation processes of IF_1_. This can be viewed as an inevitable property for IF_1_ to avoid being Maxwell’s demon that violates the second law of thermodynamics by allowing only one direction of motion in a microscopic system. If IF_1_ can dissociate from F_1_ without coupling with clockwise rotation, while IF_1_ association is tightly coupled with counterclockwise rotation, the second law of thermodynamics will be violated as Maxwell’s demon. This is evident when one considers the rotary motion of F_o_F_1_ under conditions where the torques of F_1_ and F_o_ are balanced. In IF_1_-free conditions, the rotor complex should show non-biased Brownian motion in both directions: clockwise and counterclockwise, in the F_o_F_1_ complex. Even though the rotor can cause occasional rotary steps in both directions, the mean rotational displacement does not increase. In contrast, in the presence of IF_1_, F_1_ should make a +200° rotation coupled upon the association of IF_1_. This is because ‘the tight coupling of IF_1_ association and the rotation’ means that IF_1_ association induces +200° rotation. Although the pause by IF_1_ inhibition should be long, IF_1_ should eventually dissociates from F_1_. Along the above assumption, IF_1_ is dissociated without biasing rotation. Thus, each cycle of association and dissociation of IF_1_ should cause the rotation by +200° in hydrolysis direction. After multiple events of IF_1_ association and dissociation, F_1_ undergoes unidirectionally biased rotation in hydrolysis direction. Thus, the assumption of the asymmetric coupling model should violate the second law of thermodynamics, and the reversibility of IF_1_ association/dissociation seems inevitable. For the same reason, the requirement for ADP and P_i_ for IF_1_ dissociation is also inevitable, considering that IF_1_ association is coupled with ATP hydrolysis.

This consideration would be applicable to other regulatory systems of F_o_F_1_. In addition to IF_1_, several types of inhibitory mechanisms can block the ATP hydrolysis. The ζ subunit of α-proteobacteria inhibits hydrolysis by binding to the interface of the αβ_DP_ pair in a manner similar to IF_1_^61–63^. Mycobacteria have a specific insertion sequence at the C-terminal of the α subunit called the α-extension loop^64,65^. Recent cryo-EM analysis showed that the α-extension loop binds to a specific loop on the γ subunit, which is considered to block rotation in a counterclockwise direction^26,66^. When these inhibitory processes are coupled with the rotation of the γ subunit, the dissociation of the ζ subunit or the α-extension loop has to be coupled with rotation in the opposite direction, according to the above contention.

All the experiments described in the main text were performed at room temperature; 23 ± 2 °C for single-molecule experiments and 25 °C for solution experiments. In order to confirm that IF_1_ kinetics at the physiological temperature of bovine mitochondria (i.e., 37 °C) is essentially the same as that at room temperature, the biochemical assay of IF_1_(WT), IF_1_(Δ1-19) and IF_1_(Δ1-22) at 37 °C was performed (Supplementary Figs. 13 and 14). As a result, the trends for the residual ATPase activity at 37 °C were principally identical to that at 25 °C as described in Supplementary Fig. 8: IF_1_(WT) showed complete inhibition with almost no residual activity, whereas IF_1_(Δ1-19) and IF_1_(Δ1-22) did not completely inhibit ATPase activity. These results suggest that the principle of IF_1_ inhibition and dissociation does not differ between room temperature to physiological temperature.

Structural analyses of the *b*MF_1_-IF_1_ complex showed that IF_1_ has interactions with *b*MF_1_ via two parts: an N-terminal region with an unstructured loop and a short helix (1–20), and a central long helix (21–60) (Fig. 5a, b). The major interaction between IF_1_ and F_1_ is formed by the central long helix, which is accommodated on the C-terminal domain of the β subunit. The N-terminal region of IF_1_ wraps around the γ subunit, with evident contacts at S11 and F22 of IF_1_. Accordingly, two scenarios for the principal mechanism of IF_1_ inhibition are possible^42^. First is the mechanical hindrance of the γ subunit rotation by the N-terminal region of IF_1_. The second model assumes the prevention of β conformational transitions by the central long helix of IF_1_. The investigation of IF_1_ mutants with N-terminal truncation provides important clues to this question. We observed that IF_1_(Δ1–19) and IF_1_(Δ1–22) maintained inhibitory potency to halt the catalysis and rotation of *b*MF_1_, although these mutants were remarkably less effective than IF_1_(WT) or other mutants. IF_1_(Δ1–22) was truncated before F22, losing all residues that were in contact or close proximity to the γ subunit. Thus, our study suggests that the interaction with the γ subunit is dispensable, supporting the latter model. Our result was also supported by the research using F_1_ and IF_1_ from yeast mitochondria: deletion of all the residues preceding F17 in yeast IF_1_, corresponding to F22 in bovine IF_1_, still maintained the inhibitory capacity^67^. This result implies a universal IF_1_ inhibition mechanism beyond the species level.

In the atomic structures of *b*MF_1_-IF_1_ and *b*MF_1_-(IF_1_)_3_, hydrophobic residues in the central long helix, including Y33, form strong interactions with the C-terminal domain of the β subunit, providing most of the binding energy. The binding of IF_1_ to F_1_ is further augmented by salt-bridge formation between E30 in IF_1_ and R408 in the β_DP_ subunit. The contribution of these residues was experimentally confirmed in mutagenetic approaches^47,48^. A possible molecular mechanism for IF_1_ inhibition is that the tight binding of IF_1_ with the β subunit prevents conformational transition required for rotary catalysis. There are several highly conserved charged residues in the C-terminus of IF_1_, and the roles of these residues have not been clarified. As a preliminary trial, we conducted molecular dynamics simulation^68^ where the γ subunit is forcibly rotated in synthesis direction. The interaction between the charged residues of the γ subunit and negative charge of IF_1_ was suggested, which is reminiscent of the ionic track^69^. Based on this observation, we tested the role of these conserved charged residues by selectively substituting five residues with alanine (Supplementary Figs. 11 and 12). Except for a slight decrease in binding affinity to *b*MF_1_, no clear differences were found between IF_1_(WT) and the alanine-substitution mutant in the IF_1_ inhibition assay as well as in manipulation assay. Our experiments suggest that C-terminal charged residues have little impact on the inhibitory mechanism and the rotation-direction-dependent dissociation.

Analyses of N-terminal truncation mutants revealed the molecular mechanism of rotary-direction-dependent dissociation of IF_1_ from F_1_ (Fig. 6b). IF_1_(Δ1–22), which loses residues in contact with the γ subunit, showed a significantly high activation probability after the forcible rotation of 360°. The important finding in this mutant is that both of the clockwise and counterclockwise rotation resulted in the efficient activation from IF_1_ inhibition with almost equal probability (Fig. 5d, e). This is in contrast to the asymmetric features of IF_1_(WT), IF_1_(Δ1–7), and IF_1_(Δ1–12), which showed activation only via clockwise rotation. In the case of IF_1_(Δ1–19), the asymmetric effect of forcible rotation was retained. However, a higher activation probability is observed. These observations indicate that the N-terminal region of the central long helix plays a crucial role in rotary-direction-dependent activation, and the N-terminal short helix also contributes to this result. The truncated residues in IF_1_(Δ1–22), in addition to the 1–19 truncation, are G20, A21, and F22. Among them, F22 is bulky compared with the other residues. Further, in the crystal structures of the *b*MF_1_-IF_1_ complex, F22 is in direct contact with I16 of the γ subunit. Considering these points, F22 is likely one of the most critical residues for rotary-direction-dependent activation. Molecular dynamics simulations of the *b*MF_1_-IF_1_ complex would provide more detailed information on the molecular mechanism of rotation-direction-dependent activation.

## Methods

### Purification of *b*MF_1_ and IF_1_

F_1_-ATPase from bovine mitochondria with two mutated cysteine residues on A99 and S191 at the γ subunit and nine histidine residues (His-tag) at the N-terminus of the β subunit (hereafter referred to as *b*MF_1_) was purified as described previously^20^. IF_1_^1–60^ with the linker and mScarlet fused to the C-terminus (referred to as IF_1_(WT)) was purified as described previously^48^. The N-terminal truncation mutants and the C-terminal alanine substitution mutant of IF_1_ were prepared as follows. Fragments of the N-terminal truncated mutants, deleted in the region encoding the corresponding N-terminal region, were generated by PCR using sets of mutation primers. The sequence encoding the C-terminal alanine mutant was amplified by PCR using primers containing mutations. The sequences of the PCR primers are provided in the Source Data file. The resulting PCR products were subjected to gel electrophoresis and purification. The insert plasmid and the vector IF_1_(WT) plasmid were digested with the same restriction enzymes. The products were ligated and introduced into *Escherichia coli* JM109. The sequence of the recombinant IF_1_ plasmid was confirmed using the Fasmac sequencing service (Fasmac, Japan). The purified mutant proteins were confirmed by SDS-PAGE and MALDI-TOF/TOF mass spectrometry (Genomine. Inc., Korea or IDEA Consultants, Inc., Japan) (Supplementary Fig. 15 and Supplementary Table 1).

### Solution experiment

The ATPase activity of *b*MF_1_ was monitored as the rate of NADH oxidation^48^. The basal buffer contained 50 mM HEPES-KOH (pH 7.5), 50 mM KCl, 2 mM MgCl_2_, and an ATP-regenerating system (0.2 mg/mL pyruvate kinase and 2.5 mM phosphoenolpyruvate) supplemented with 0.2 mM NADH and 50 μg/mL lactate dehydrogenase, as described previously^48^. Experiments were performed at 25°C, unless otherwise indicated, using a V-660 (JASCO, Tokyo, Japan) UV/VIS spectrophotometer equipped with a peltier-type temperature controller (ETCS-761). ATP hydrolysis by *b*MF_1_ was initiated by adding purified *b*MF_1_ to the basal buffer containing 1 mM ATP. For the reaction to reach a steady state, we waited for 180 s before injecting IF_1_ into the reaction mixture. After the IF_1_ injection, the rate of ATPase activity changed. Inhibition by IF_1_ was quantified by estimating the apparent rate constants for IF_1_ (${k}_{{inhibition}}^{{app}}$), which were determined by fitting the decay using the following equation:

1 $$y\left(t\right)-{y}_{0}={V}_{{{\infty }}}t+\frac{{V}_{0}-{V}_{{{\!\infty }}}}{{k}_{{inhibition}}^{{app}}}\left\{1-{{\exp }}\left(-{k}_{{inhibition}}^{{app}}t\right)\right\}$$

where $y\left(t\right)$ and ${y}_{0}$ are the absorbance values at the time *t* and 0 after IF_1_ injection into the solution cuvette, respectively, and ${V}_{0}$ and ${V}_{\infty }$ are the initial and final reaction rates, respectively. Fitting was performed using the time course of NADH absorbance at 2 s after IF_1_ addition. Because no exponential decay was observed in the time course of IF_1_(Δ1–19) and IF_1_(Δ1–22) inhibition (Supplementary Figs. 8g, 13c, e), curve fitting using Eq. (1) was not performed. Residual ATPase activity was measured by comparing the activity just before IF_1_ addition and 350 s after IF_1_ addition. In Supplementary Figs. 8 and 11, values were plotted over intermediate or higher IF_1_ concentrations (${K}_{M}^{I{F}_{1}}$) to see equilibrium at saturating conditions.

The sequential IF_1_ inhibition was analyzed using the following reaction scheme:

$${F}_{1}+I{F}_{1}\,\begin{array}{c}\mathop{\to }\limits^{\,{k}_{{on}}}\\ \mathop{\leftarrow }\limits_{{k}_{{off}}}\end{array}\,{F}_{1}\cdot I{F}_{1}\,\mathop{\to }\limits^{{k}_{{lock}}}\,{F}_{1}\cdot I{F}_{1}^{{lock}}$$

where ${F}_{1}\cdot I{F}_{1}^{{lock}}$ represents the inactive state, and ${F}_{1}\cdot I{F}_{1}$ is the intermediate state that is still catalytically active. ${k}_{{on}}$ and ${k}_{{off}}$ represent the rate constants of association and dissociation, respectively. ${k}_{{lock}}$ is the rate constant of isomerization to the locked state. According to this scheme, ${k}_{{inhibition}}^{{app}}$ can be expressed as follows:

2 $${k}_{{inhibition}}^{{app}}=\frac{{k}_{{lock}}\left[I{F}_{1}\right]}{{K}_{M}^{I{F}_{1}}+\left[I{F}_{1}\right]}$$

3 $${K}_{M}^{I{F}_{1}}\equiv \frac{{k}_{{off}}+{k}_{{lock}}}{{k}_{{on}}}$$

which shows a typical hyperbolic relationship.

### Rotation assay of *b*MF_1_

For the rotation assay, *b*MF_1_ was immobilized on the glass surface, and magnetic beads were attached to the two cysteine residues of the γ subunit (γA99C and γS191C) through a biotin-avidin interaction. The protocol was as follows^20^. A flow chamber (~5 μL) was prepared with two coverslips (18 × 18 mm^2^ at the top and 24 × 32 mm^2^ at the bottom; Matsunami Glass) with double-sided tape (7602 #25, Teraoka) as a spacer. Purified *b*MF_1_ (~500 pM) in observation buffer (50 mM HEPES-KOH (pH 7.5), 50 mM KCl, and 2 mM MgCl_2_) was gently introduced into the flow chamber and incubated for 5–10 min. After washing unbound *b*MF_1_ with more than 50 μL of observation buffer containing ~10 mg/mL BSA, streptavidin-coated magnetic beads (GE Healthcare), which were pre-centrifuged to allow the use of relatively small beads (~300 nm), were introduced into the flow chamber. Unbound beads were washed with more than 50 μL of observation buffer containing the prescribed concentrations of ATP and/or ADP/P_i_. Rotations of the magnetic beads were observed using a phase-contrast microscope (IX-70, Olympus) with a 100× objective lens at a recording rate of 30 fps. The rotation assay was performed at 23 ± 2 °C. The ATP-regenerating system (0.2 mg/mL pyruvate kinase and 2.5 mM phosphoenolpyruvate) was added to the solution mixture, except when ADP was used.

To observe the IF_1_ inhibitory state, we identified rotating molecules among the particles attached to the coverslip in the IF_1_-free solution. Next, a solution containing IF_1_ in ATP solution or ATP synthesis buffer (100 μM ATP, 100 μM ADP, and 1 mM P_i_) was infused into the flow chamber. After rotations for several tens of seconds, all *b*MF_1_ molecules stopped rotation (Fig. 2a).

### Manipulation with magnetic tweezers

Magnetic tweezers, consisting of two sets of electromagnets, were equipped onto the microscope stage and controlled by custom-made software^20,58^. Rotations were imaged at 30 fps using a progressive-scan camera (FC300M, Takex, Kyoto, Japan), which allowed real-time manipulation with magnetic tweezers. Movies were stored on a computer as AVI files and analyzed using custom-made software.

### Kinetic analysis of ADP inhibition

Rotations in the IF_1_-free solution were recorded for more than 10 min per molecule (Supplementary Fig. 1). Under these conditions, ATP binding occurs for less than 1 ms and cannot be detected as a pause of rotations with magnetic beads at a recording rate of 30 fps. However, *b*MF_1_ showed frequent transient pauses; we collected the data from all pauses longer than 1 s^31^, and rotary traces between pauses longer than 1 s were defined as rotations. Distributions of pausing times before the onset of rotations were fitted by a double exponential function, $y={N}_{{sp}}{{\exp }}(-t/{\tau }_{{sp}})+{N}_{{lp}}{{\exp }}(-t/{\tau }_{{lp}})$, where ${N}_{{sp}}$ and ${N}_{{lp}}$ are constants, and ${\tau }_{{sp}}$ and ${\tau }_{{lp}}$ are time constants for the short and long pause, respectively. A relatively short pause (sp) corresponds to decelerated P_i_ release, as previously reported^31,70^. The long pause (lp) corresponds to the ADP-inhibited form. These results showed that the time scale for ADP inhibition was 10-30 s under the various conditions tested. Distributions of rotating times before lapsing into the pauses were fitted by a single exponential function, $y={N}_{{rot}}{{\exp }}\left(-t/{\tau }_{{rot}}\right)$, where ${N}_{{rot}}$ is a constant and ${\tau }_{{rot}}$ is a time constant for rotations. In Supplementary Fig. 1, the histograms include most of the total data; the remaining data are not shown in the figure for clarity. All data are provided in the Source Data.

### Analysis of IF_1_ inhibition

The experimental procedure was described in the main text (see also Fig. 2a). Rotating time (Supplementary Figs. 2, 3, 6, 10, and 12) was defined as the period of time from the completion of solution exchange until when the molecules fell into IF_1_ inhibition. Probability plots versus rotating time were fitted with a single-exponential decay function to estimate the mean rotation time, ${\tau }_{{rot}}^{I{F}_{1}}$. The figures include most of the total data; the remaining data are not shown in the figure for clarity. All data are provided in the Source Data. A *pause* was defined as a pause state for more than 1 s. Except for IF_1_(Δ1–19) and IF_1_(Δ1–22), most of the pauses showed extremely long pausing states of more than 480 s, corresponding to IF_1_ inhibition. A few traces showed relatively short pausing states of ~20 s. They were attributable to ADP inhibition because the timescales of these pauses were mostly identical to the values estimated from the IF_1_-free solution experiment (see Supplementary Fig. 1). All of them spontaneously recovered active rotation and eventually fell into IF_1_ inhibition without exception. For IF_1_(Δ1–19) and IF_1_(Δ1–22), rotations were frequently recovered without manipulation, suggesting a reversible inhibition/dissociation mechanism. The histogram of pausing time was fitted by single-exponential decay function for IF_1_(Δ1–19) to estimate the mean duration time, ${\tau }_{{pause}}^{I{F}_{1}}$, 254 s. For IF_1_(Δ1–22) analysis, the double-exponential decay function was applied to fit the histogram, estimating ${\tau }_{{lp}}^{I{F}_{1}}$ and ${\tau }_{{sp}}^{I{F}_{1}}$. Although the underlying molecular mechanism corresponding to ${\tau }_{{sp}}^{I{F}_{1}}$ is unclear, we selected ${\tau }_{{lp}}^{I{F}_{1}}$ for IF_1_(Δ1–22) inhibitory states.

### Reporting summary

Further information on research design is available in the Nature Portfolio Reporting Summary linked to this article.

## Supplementary information

Supplemental information

Peer review file

Reporting Summary

## Data Availability

The structural information used for Figs. 1b, 5a, b, and 6b is accessible in PDB accession number 2v7q [10.2210/pdb2v7q/pdb]. Source data are provided with this paper.

## Source data

Source Data
