# Supplementary material for: Molecular mechanism on forcible ejection of ATPase inhibitory factor 1 from mitochondrial ATP synthase
Source: Nat Commun. 2023 Mar 31;14:1682. doi: 10.1038/s41467-023-37182-9 (PMC10066207; doi:10.1038/s41467-023-37182-9)
Supplement: Supplementary file 1 — Supplemental information [file 41467_2023_37182_MOESM1_ESM.pdf]

Supplementary information for

Molecular mechanism on forcible ejection of ATPase inhibitory factor 1 from mitochondrial ATP synthase

Ryohei Kobayashi[a,b], Hiroshi Ueno[a], Kei-ichi Okazaki[b,c], Hiroyuki Noji[a,*]

This PDF file includes:

- Supplementary Note
  - Selection of the molecules in the manipulation experiments
  - Probability of spontaneous reactivation during manipulation
  - Molecular dynamics (MD) simulation
- Supplementary Figures 1 to 15
- Supplementary Tables 1 to 6
- Supplementary References

[a] *Department of Applied Chemistry, Graduate School of Engineering, The University of Tokyo, Bunkyo-ku, Tokyo 113-8656, Japan*

[b] *Research Center for Computational Science, Institute for Molecular Science, Okazaki, Aichi 444-8585, Japan*

[c] *The Graduate University for Advanced Studies, SOKENDAI, Okazaki, Aichi 444-8585, Japan*

*Corresponding author: Hiroyuki Noji

Phone number: +81-3-5841-7252

Email: hnoji@g.ecc.u-tokyo.ac.jp

Supplementary Note

Selection of the molecules in the manipulation experiments

To distinguish IF_1 inhibition from ADP inhibition, we employed the following method in the manipulation experiments. For the forcible 360° manipulation experiment (Figs. 3b, 5d, 5e and Supplementary Figs. 5 and 12d), we waited for 480 s after confirming a pause because IF_1 inhibition was irreversible, and *b*MF_1 did not recover rotation within the observation period (*e.g.*, Supplementary Fig. 2a). In contrast, ADP inhibition was reversible and *b*MF_1 spontaneously recovered rotation. It is noted that the difference between IF_1 inhibition and ADP inhibition is not only in the length of the pause time but also in the activation probability by counterclockwise manipulation. Pauses by ADP inhibition observed in IF_1-free solution can be almost activated by the stall at +80° in the counterclockwise direction for 2 or 5 s (Supplementary Fig. 7, triangles). In contrast, IF_1 inhibition, defined as pauses longer than 480 s under saturating IF_1 conditions, was not activated by the same manipulation (Supplementary Fig. 7, squares). Thus, IF_1-inhibited states were confirmed as follows; after confirming re-inactivation by IF_1 in solution, we stalled the targeted molecules at +80° in the clockwise direction before each trial. We analyzed the molecule that did not show reactivation by the abovementioned manipulation.

In the "stall-and-release" experiment, we selectively analyzed the molecule that showed activation by the forcible 360° clockwise rotation. Thus, the maximum probability shown in the figures (*e.g.*, Fig. 4c) was 100%, whereas approximately 60% of all molecules are subject to analysis in the forcible 360° manipulation. Upon repeated manipulation with magnetic tweezers, molecules sometimes showed unusual fluctuations, which may reflect the unstable states of the molecules or their detachment from the coverslip (Supplementary Table 6). Such molecules could not be reactivated by the forcible 360° clockwise rotation. Contamination of these molecules in the analysis reduces the probability of reactivation. We concluded that reliable data could not be obtained if such disordered molecules were subjected to further experiments. For molecules that did not resume active rotation after the forcible 360° clockwise rotation, we completed manipulation of the molecules and searched for a new molecule.

Probability of spontaneous reactivation during manipulation

In contrast to IF_1(WT), IF_1(Δ1–7) and IF_1(Δ1–12), IF_1(Δ1–19) and IF_1(Δ1–22) did not completely stop the rotation of *b*MF_1. Given the inherent pause time of these two mutants, 250 s and 60 s, respectively (Supplementary Fig. 10k and 10n), we selectively manipulated pauses that lasted for longer than 100 s for IF_1(Δ1–19) and 60 s for IF_1(Δ1–22).

Inhibitory states by IF_1(Δ1–22) were reactivated by manipulation of 360° at the rate of 0.5 rps, *i.e.*, 2 s per manipulation. To exclude the possibility that the rotations resumed independently of

the 2 s manipulation, we performed the following calculation. The average pause time by IF_1(Δ1–22) was 60 s, although short pauses of only 2 s on average were also observed. As mentioned above, we waited for 60 s to selectively manipulate the long pauses after confirming the pauses. Thus, the impact of short pauses on this analysis was negligible. The probability of these pauses ending in 2 s is calculated using the following equation:

$$100 \times \frac{\int_0^2 \exp\left(-\frac{t}{60}\right) dt}{\int_0^\infty \exp\left(-\frac{t}{60}\right) dt} = 3\%$$

Therefore, the probability of simultaneous reactivation was so low that we concluded that the reactivation of rotation was induced by the manipulation itself.

Molecular dynamics (MD) simulation

The MD simulation of the IF_1-bound F_1 was performed based on the previous work [1] with some modifications. The initial structure was taken from the IF_1-bound crystal structure of *b*MF_1 (PDB: 2v7q) [2]. Residues 23–510 were used for the α subunits, 9–478 were used for the β subunits, and all residues were used for the γ subunit and IF_1. MODELER [3] was used to model the structure of missing residues. The δ and ε subunits were not considered. The bound nucleotides and Mg^{2+} ions were retained as in the crystal structure. Crystal water molecules were also retained unless they clearly overlapped with replaced ligands.

The modeled F_1 was solvated with TIP3P water [4] in a rectangular box such that the minimum distance to the edge of the box was 11 Å. Then, 150 mM KCl was then added to neutralize the system. The total number of atoms is ~320,000. The Amber ff14SB force field was used for the protein [5], with the previously developed parameters for ATP and ADP [6], and for P_i [1]. The system was energy minimized and equilibrated under isothermal–isobaric (NPT) conditions with Ewald electrostatics and restraints on all heavy atoms in the protein for 500 ps, and subsequently, with restraints on only Cα atoms for 1 ns. After the equilibration, production runs were performed with restraints on the Cα atoms of the 10 N-terminal residues of each of the three β subunits, mimicking the role of His-tag anchoring in single-molecule experiments. NAMD 2.12 was used for the MD simulation with periodic boundary condition[7]. Langevin dynamics with 1 ps^{-1} damping coefficient was used for temperature control at 310 K, and the Nose–Hoover Langevin piston was used for pressure control at 1 atm [8]. The torque-applying simulations to the γ subunit in the clockwise direction were performed at the rate of 1°/ns of γ rotation as in the previous studies [1,9].

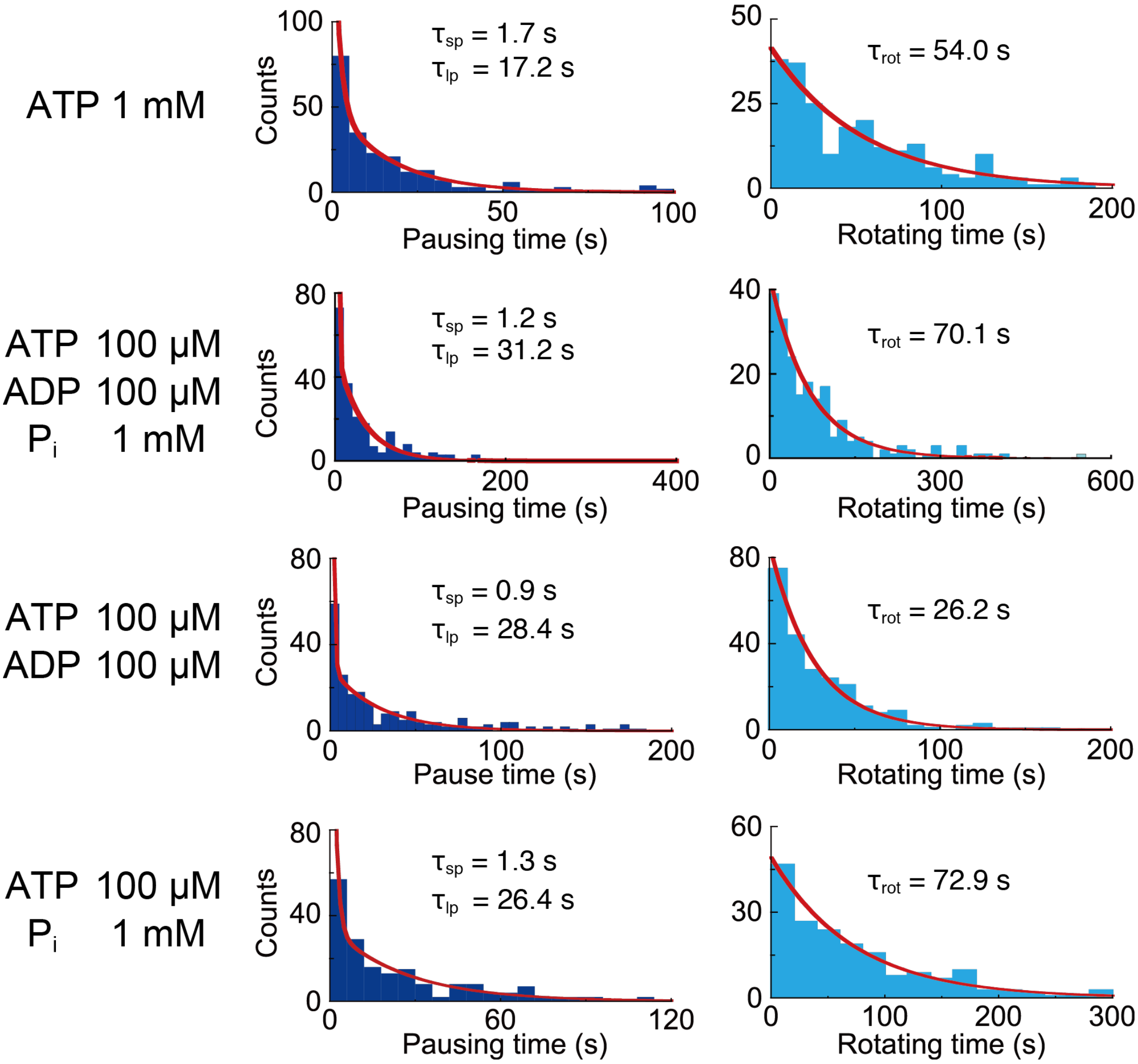

Supplementary Figure 1. Kinetic analysis of ADP-inhibited form in *b*MF$_1$ in the absence of IF$_1$ at indicated conditions.

(*Left*) Distribution of pause time before the onset of rotations (*N* = 196-238 pauses, 7-11 molecules). Pauses over 1 s were collected. τ_{sp} and τ_{lp} were estimated from the fitting of the histogram by a double exponential decay function, $y = N_{sp}\exp(-t/\tau_{sp}) + N_{lp}\exp(-t/\tau_{lp})$, as previously described [10]. (*Right*) Distribution of rotation time before lapsing into pauses (*N* = 186-232 traces, 7-11 molecules). τ_{rot} was estimated from the fitting of the histogram by a single exponential decay function. When ADP was included in the mixture, the ATP-regeneration system was omitted. Source data are provided in the Source Data file.

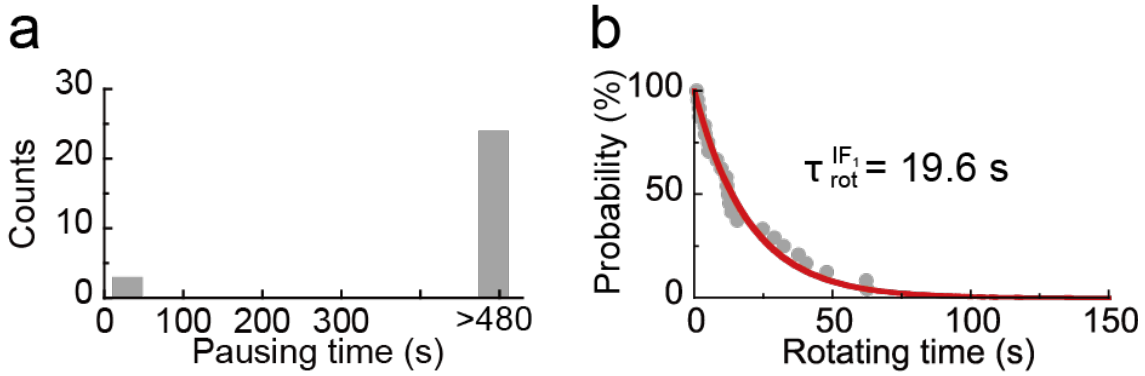

Supplementary Figure 2. Rotations and pauses of *b*MF$_1$ with 3 µM IF$_1$ at 1 mM ATP.

(a) The histogram of pause time (N = 27 pauses). Durations of all pauses over 1 s were plotted. 24 of 27 pauses showed extremely long pauses over 480 s, corresponding to IF$_1$-inhibited pauses, whereas the other 3 pauses showed relatively short pauses, less than 20 s, which were attributable to ADP-inhibited form (=17 s in the absence of IF$_1$, shown in Supplementary Fig. 1). All of the ADP-inhibited pauses spontaneously resumed active rotations and finally fell into IF$_1$-inhibited states. (b) Distribution of rotation time required to reach IF$_1$-inhibited states after IF$_1$ addition. The solid line represents the fitting curve of an exponential decay function (N = 24 traces), which estimates the mean rotation time. Source data are provided in the Source Data file.

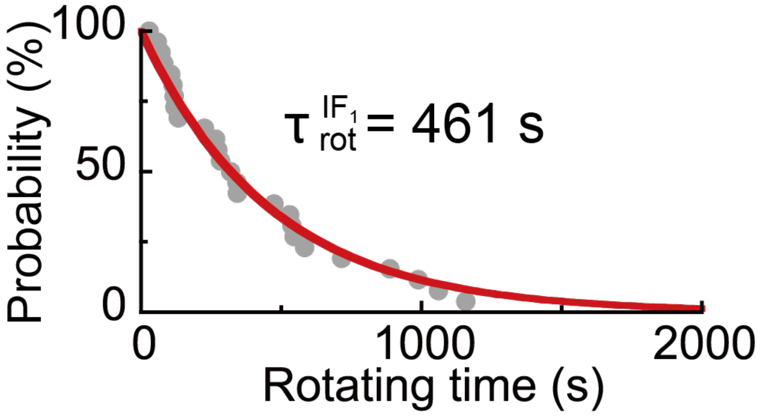

Supplementary Figure 3. Rotation time of *b*MF_1 to reach IF_1-inhibited states at 100 nM ATP and 5 µM IF_1.

Plots were fitted with a single-exponential decay function (N = 26 pauses). Values represent fitting parameters. Source data are provided in the Source Data file.

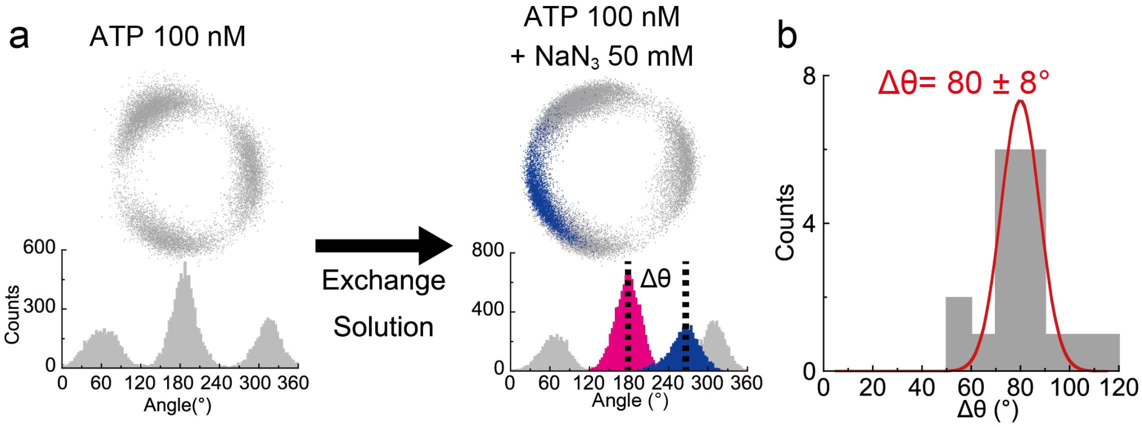

Supplementary Figure 4. Stall positions by NaN_3.

(a) Experimental procedure. After observing the ATP-binding waiting dwell at 100 nM ATP, 50 mM NaN_3 with 100 nM ATP was introduced into the reaction chamber. Blue data points represent the positions blocked with NaN_3. (b) The angular distance ($\Delta\theta$) of NaN_3 inhibition from the left-side ATP-binding waiting dwell (pink) (N =18 pauses). Values represent mean ± SD estimated from Gaussian fitting of the plots. Source data are provided in the Source Data file.

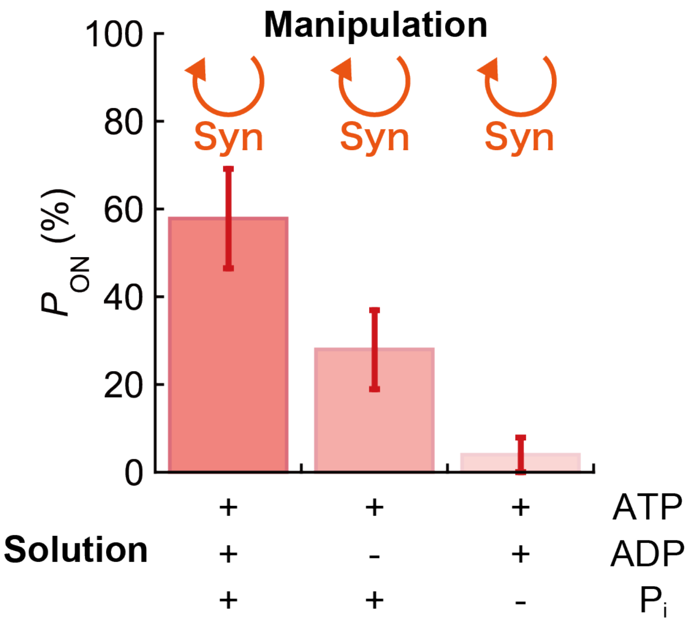

Supplementary Figure 5. Reactivation probability of IF_1-inhibited *b*MF_1 after clockwise manipulation.

Experiments were conducted under 100 µM ADP and/or 1 mM P_i with 100 µM ATP. Values represent reactivation probability (P_{ON}) ± SD. P_{ON} was defined as the probability of an ON event against total molecules. The SD of P_{ON} is given as $\sqrt{P_{ON}\,(100 - P_{ON})/N}$, where N is the number of total molecules (N = 19-25 molecules). Source data and the exact number of molecules in each data point are provided in the Source Data file.

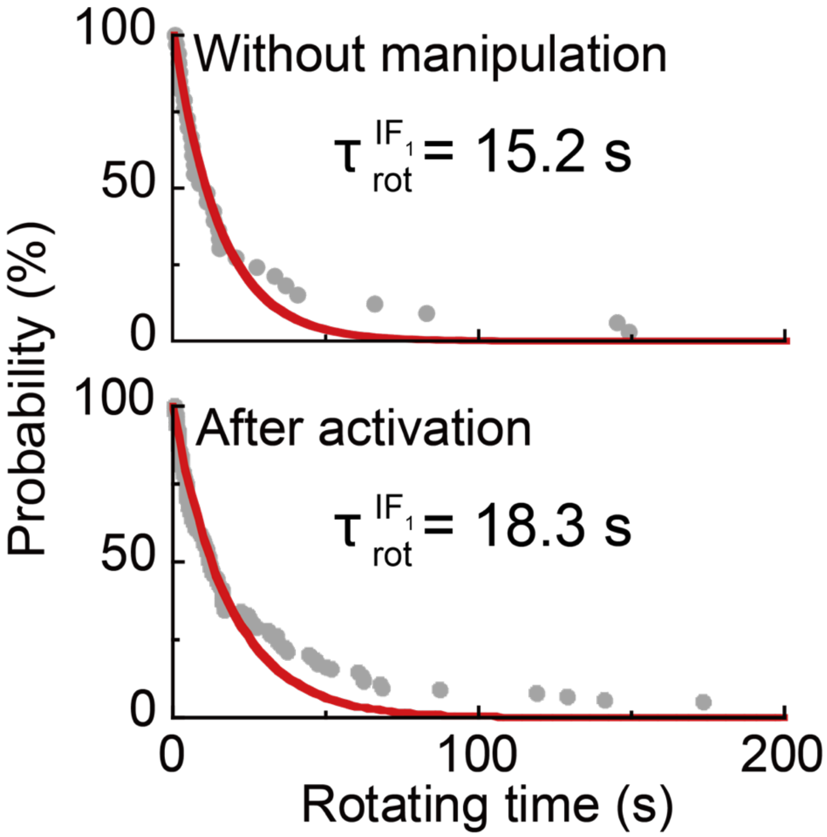

Supplementary Figure 6. Rotation time analysis.

(*Upper*) Rotation time before inhibition after solution exchange (N = 33 traces). Molecules in the active state under IF_1-free conditions were analyzed. Rotation time was defined as the time from completion of solution exchange until the molecules fell into IF_1 inhibition. (*Lower*) Rotation time until re-inactivation of activated F_1 molecules (N = 104 traces). The IF_1-inhibited *b*MF_1 was activated by magnetic tweezers. Experiments were conducted under 100 μM ATP, 100 μM ADP, 1 mM P_i and 3 μM IF_1. Plots were fitted with a single-exponential decay function. Values represent fitting parameters. Source data are provided in the Source Data file.

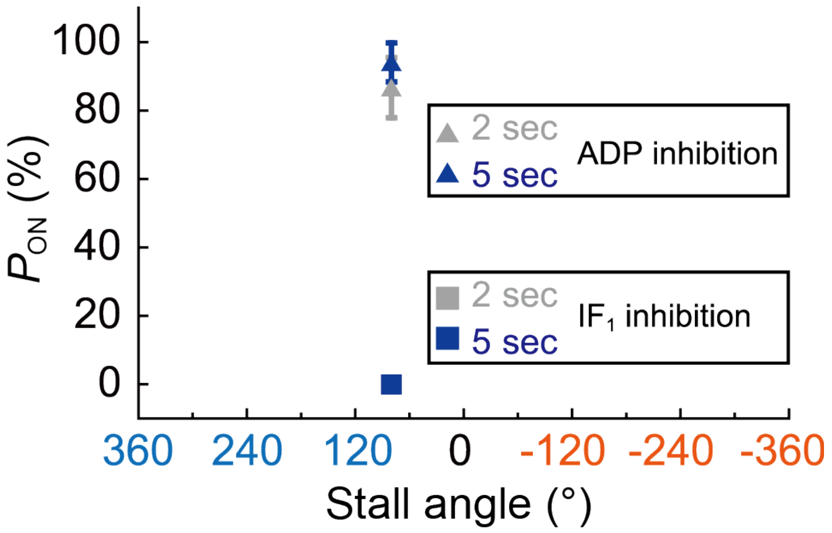

Supplementary Figure 7. The stall-and-release experiment of IF$_1$ inhibition and ADP inhibition at 80° to the counterclockwise direction from the initial pause angle.

Gray and blue symbols represent the stall time of 2 s and 5 s, respectively. Triangles represent the manipulation of ADP-inhibited states in the IF$_1$-free (100 μM ATP, 100 μM ADP, and 1 mM P_i) condition. Squares represent the manipulation of IF$_1$-inhibited states with 3 μM IF$_1$ under 100 μM ATP, 100 μM ADP, and 1 mM P_i. Notably, trials of 2 and 5 s stalls showed no activation (0%), and the gray and blue squares overlap in this figure. Manipulation of IF$_1$-inhibited states was conducted after confirming a pause longer than 480 s in the solution containing IF$_1$. IF$_1$ inhibition can be successfully discriminated from ADP inhibition because IF$_1$-inhibited states were not reactivated by this manipulation. Each data point was obtained from 15 to 18 trials using 3 to 7 molecules. Values represent reactivation probability (P_{ON}) ± SD. P_{ON} was defined as the probability of an ON event against total molecules. The SD of P_{ON} is given as $\sqrt{P_{ON}\,(100-P_{ON})/N}$, where N is the number of total trials. Source data and the exact number of molecules in each data point are provided in the Source Data file.

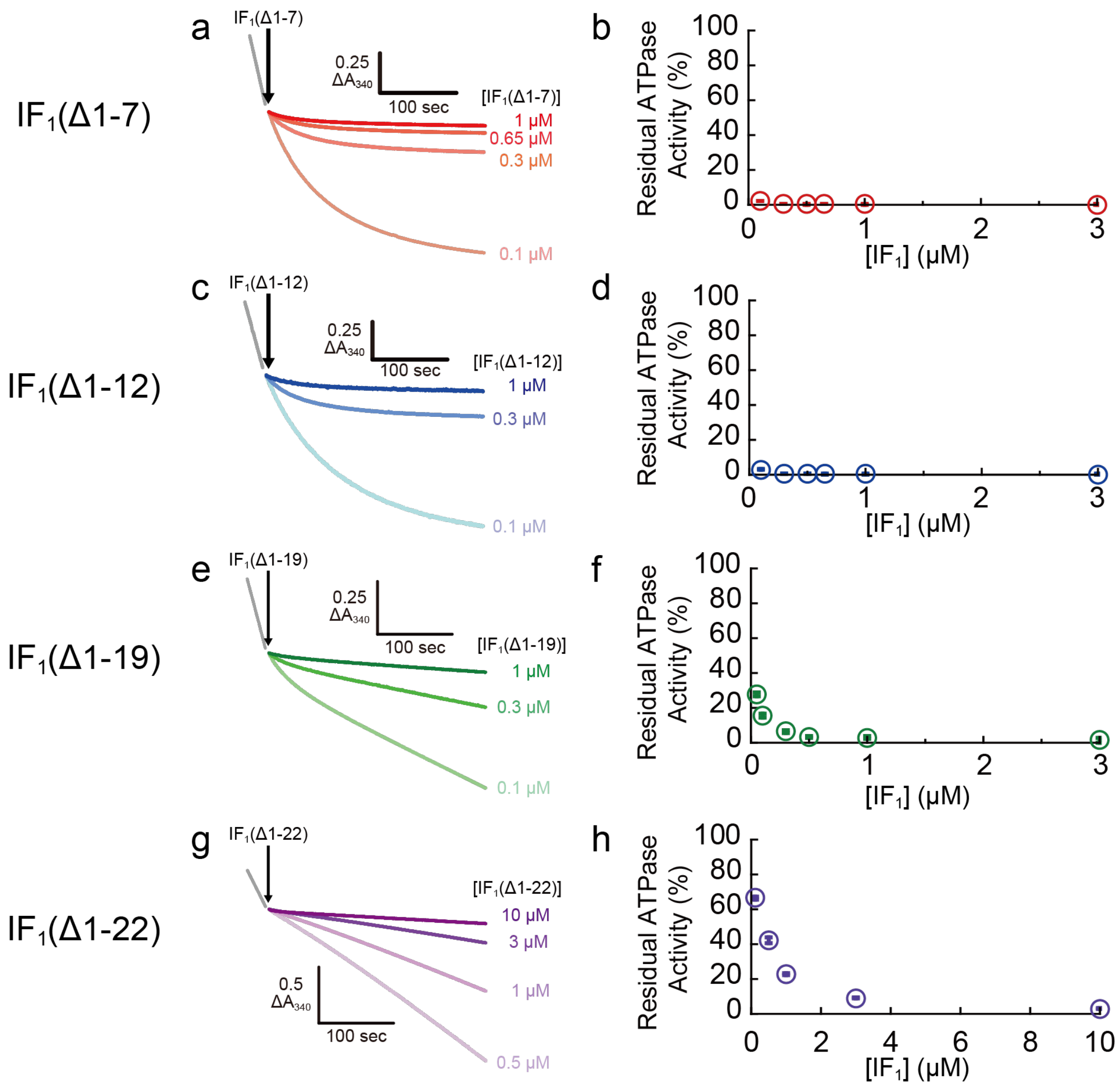

Supplementary Figure 8. Biochemical analysis of N-terminal truncated mutants of IF_1.

(a, c, e, g) Time courses for IF_1-inhibited *b*MF_1 at 1 mM ATP and the indicated concentrations of (a) IF_1(Δ1-7) (red), (c) IF_1(Δ1-12) (blue), (e) IF_1(Δ1-19) (green) and (g) IF_1(Δ1-22) (purple). The final concentration of *b*MF_1 was 10 nM. (b, d, f, h) Residual ATPase activity versus [IF_1] at the end of the measurement (350 s after IF_1 injection). Circles and error bars represent the mean value and SD, respectively (N = 3 for each measurement). Source data are provided in the Source Data file.

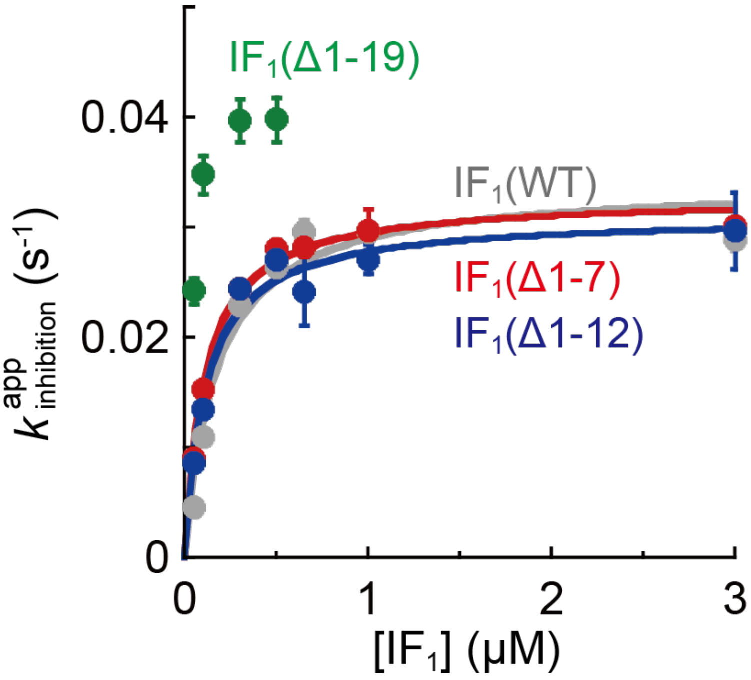

Supplementary Figure 9. $k^{app}_{inhibition}$ **plotted against [IF$_1$].**

$k^{app}_{inhibition}$ was determined from the fitting of the time course by Eq. (1), as described in the main text. Solid lines represent the fitting curve of Eq. (2). Fitting in IF$_1$(Δ1-19) was not performed because IF$_1$(Δ1-19) is a reversible association/dissociation inhibition, and Eq. (2) is not appropriate for fitting. The determined fitted parameters are summarized in Supplementary Table 1. Data fitting was conducted from 2 s after IF$_1$ injection. Circles and error bars represent the mean value and SD, respectively (*N* = 3 for each measurement). Data from IF$_1$(WT) were taken from our previous paper [11] for comparison. Source data are provided in the Source Data file.

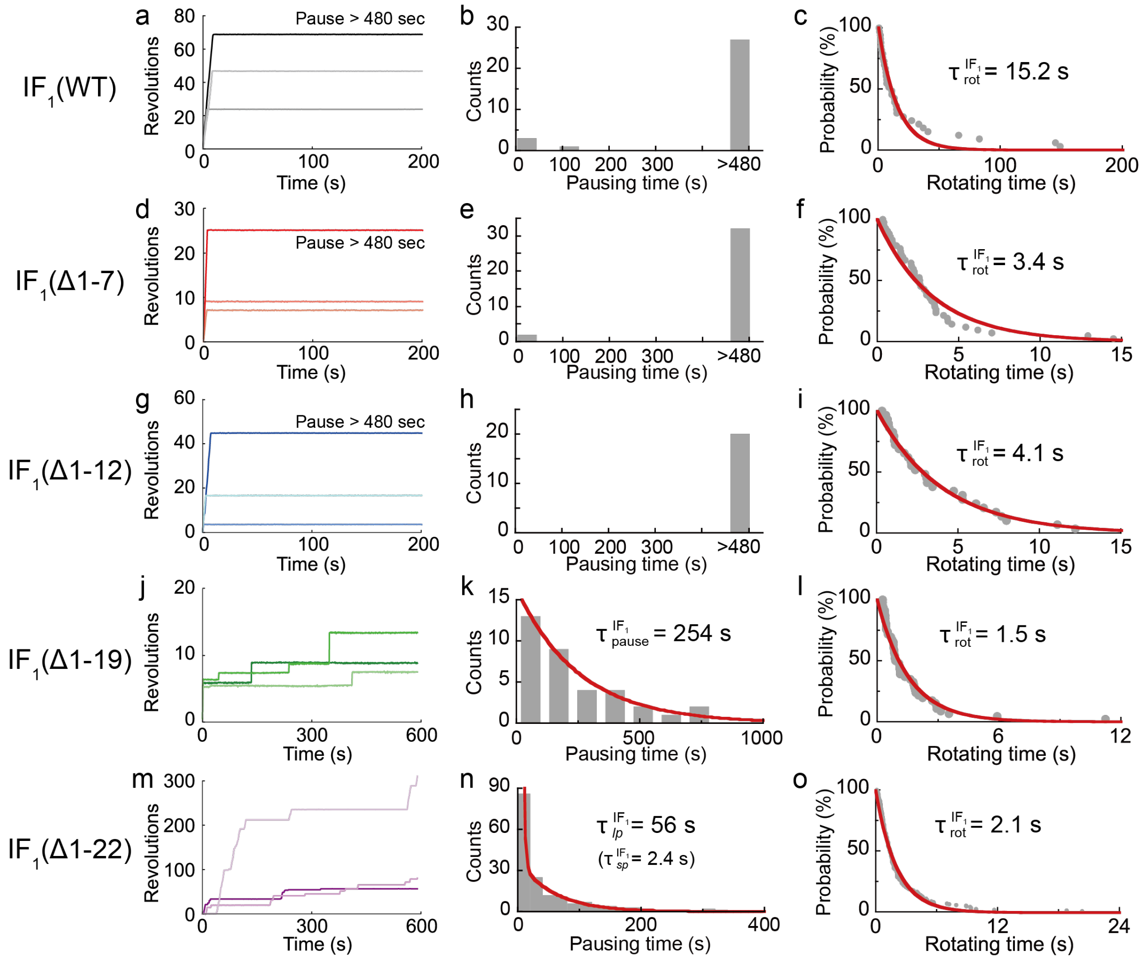

Supplementary Figure 10. Single-molecule analysis of *b*MF$_1$ in rotations and inhibitions by (a-c) IF$_1$(WT), (d-f) IF$_1$(Δ1-7), (g-i) IF$_1$(Δ1-12), (j-l) IF$_1$(Δ1-19), and (m-o) IF$_1$(Δ1-22).

(a, d, g, j, m) Typical time courses of *b*MF$_1$ rotations with 3 μM IF$_1$. Experiments were conducted under 100 μM ATP, 100 μM ADP, and 1 mM P$_i$. (b, e, h, k, n) The histogram of pause time (N = 31, 34, 20, 35, 167 pauses, respectively). Durations of all pauses over 1 s were plotted. The solid line in k represents the fitting curve of an exponential decay function that estimates the mean duration of the pause time, $\tau_{pause}^{IF_1}$. The solid line in n represents the fitting curve of a double exponential decay function, $y = N_{sp}^{IF_1}\exp(-t/\tau_{sp}^{IF_1}) + N_{lp}^{IF_1}\exp(-t/\tau_{lp}^{IF_1})$, where $N_{sp}^{IF_1}$ and $N_{lp}^{IF_1}$ are constants and $\tau_{sp}^{IF_1}$ and $\tau_{lp}^{IF_1}$ are the time constants ($\tau_{sp}^{IF_1} < \tau_{lp}^{IF_1}$), respectively. (c, f, i, l, o) The survival probability plot of rotation time required to reach IF$_1$-inhibited states after IF$_1$ addition (N = 33, 42, 29, 46, 176 traces, respectively). The solid line represents the fitting curve of an exponential decay function to estimate the mean rotation time, $\tau_{rot}^{IF_1}$. Source data are provided in the Source Data file.

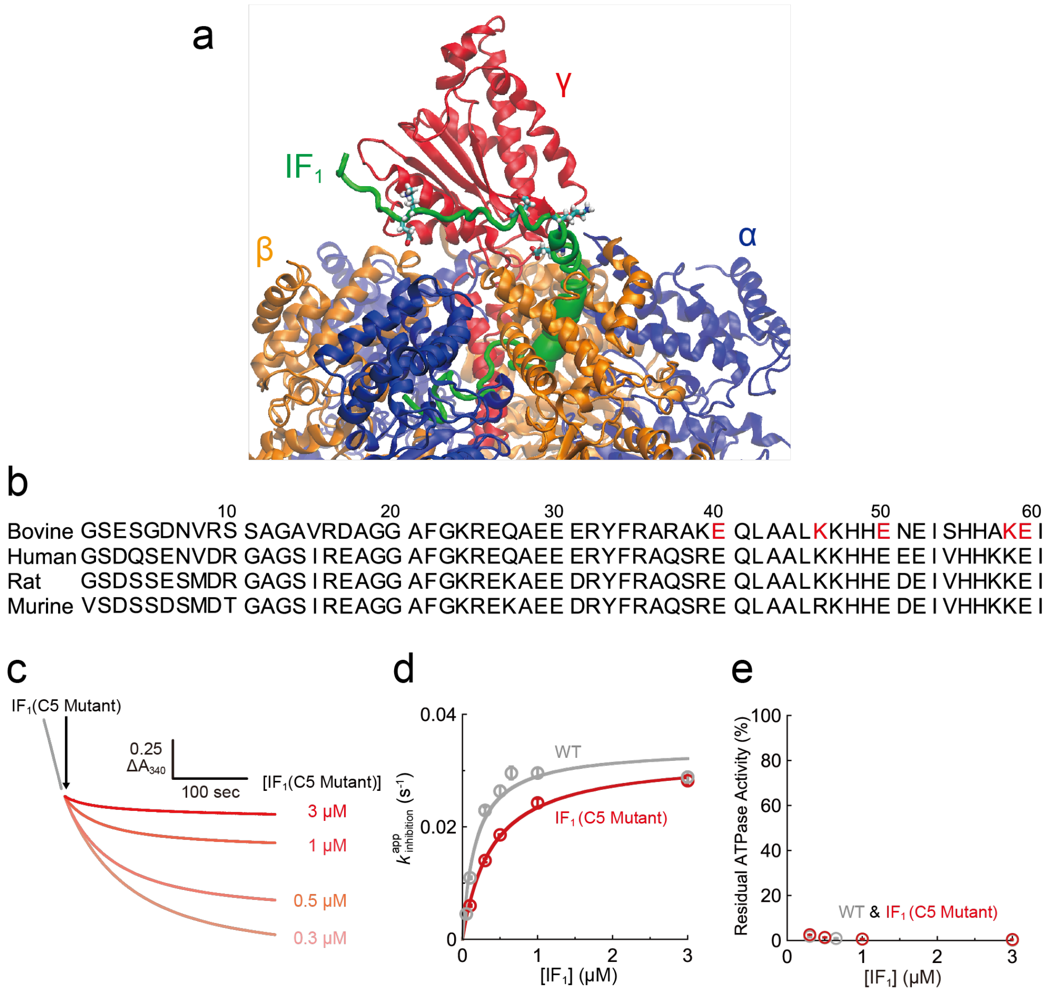

Supplementary Figure 11. Biochemical analysis of IF$_1$(C5 Mutant).

(a) A snapshot from the MD simulation at 200 ns. The IF$_1$ residues (E40, K46, E50, K58, and E59), which interact with the charged residues of γ subunit, are represented by stick form. In this figure, α, β, γ and IF$_1$ are colored by blue, orange, red, and green, respectively. (b) Alignment of amino acid sequences of mammal IF$_1$s with reference to the previous paper [12]. The IF$_1$(C5 Mutant) includes five alanine substitutions: E40A, K46A, E50A, K58A, and E59A. (c) Time courses for IF$_1$-inhibited *b*MF$_1$ at 1 mM ATP and the indicated concentrations of IF$_1$(C5 Mutant) in solution experiment (N = 3 for each measurement). (d) $k_{inhibition}^{app}$ plotted against [IF$_1$]. $k_{inhibition}^{app}$ was determined from the fitting of the time course by Eq. (1). Solid line represents the fitting curve of Eq. (2). The determined fitted parameters are summarized in Supplementary Table 1. Data fitting was conducted from 2 s after IF$_1$ injection. (e) Residual ATPase activity versus [IF$_1$] at the end of the measurement (350 s after IF$_1$ injection). Notably, the residual ATPase activity was less than 5% in all plots, and they mostly overlap in this figure. In (d) and (e), circles and error bars represent the mean value and SD, respectively (N = 3 for each measurement). Source data are provided in the Source Data file.

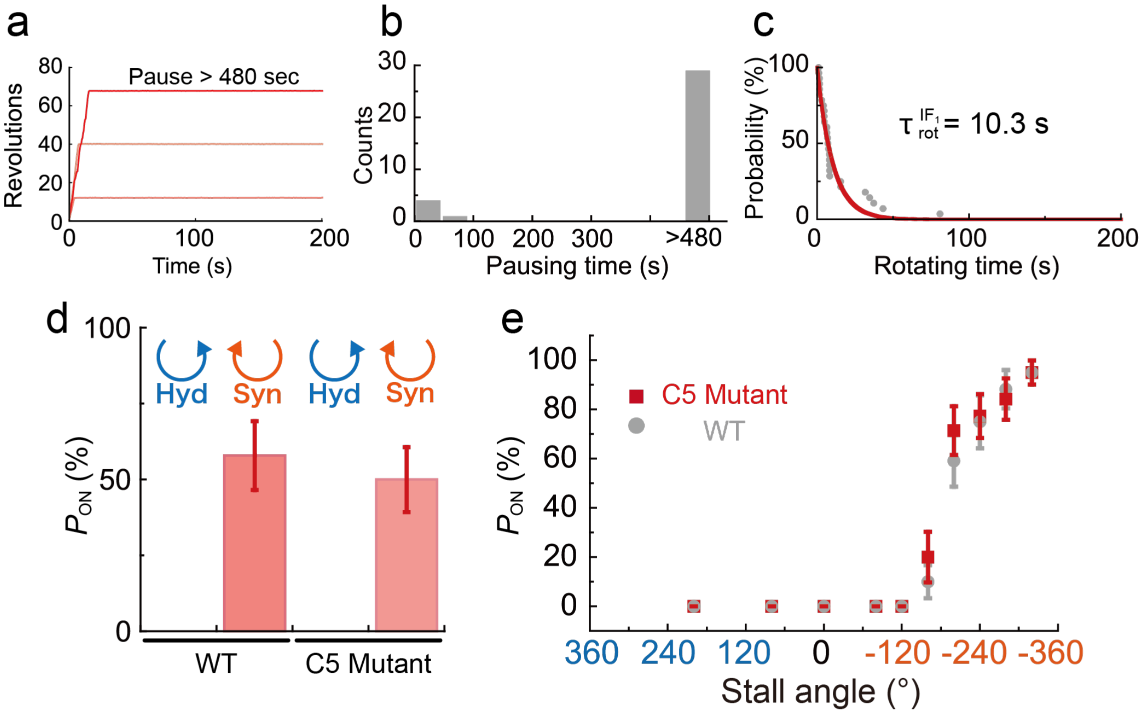

Supplementary Figure 12. Single-molecule analysis of *b*MF₁ in rotations and inhibitions by IF₁(C5 Mutant).

(a) Typical time courses of *b*MF₁ rotations with 30 µM IF₁(C5 Mutant). Experiments were conducted under 100 µM ATP, 100 µM ADP, and 1 mM Pᵢ. (b) The histogram of pause time (*N* = 34 pauses). Durations of all pauses over 1 s were plotted. 29 of 34 pauses showed extremely long pauses over 480 s, corresponding to IF₁-inhibited pauses, whereas the other 5 pauses showed relatively short pauses. (c) Distribution of rotation time required to reach IF₁-inhibited states after IF₁ addition. The solid line represents the fitting curve of an exponential decay function (*N* = 28 traces) that estimates the mean rotation time. (d) Reactivation probability of IF₁-inhibited *b*MF₁ after manipulation. Values represent reactivation probability (P_{ON}) ± SD. P_{ON} was defined as the probability of an ON event against total molecules. The SD of P_{ON} is given as $\sqrt{P_{ON}\,(100 - P_{ON})/N}$, where N is the number of total molecules (*N* = 22 molecules). (e) Angle dependence of reactivation probability with the stall time of 2 s. Each data point was obtained from 10 to 79 trials using 4 to 26 molecules. Values represent reactivation probability (P_{ON}) ± SD. P_{ON} was defined as the probability of an ON event against total trials. The SD of P_{ON} is given as $\sqrt{P_{ON}\,(100 - P_{ON})/N}$, where N is the number of total trials. Source data and the exact number of trials in each data point are provided in the Source Data file.

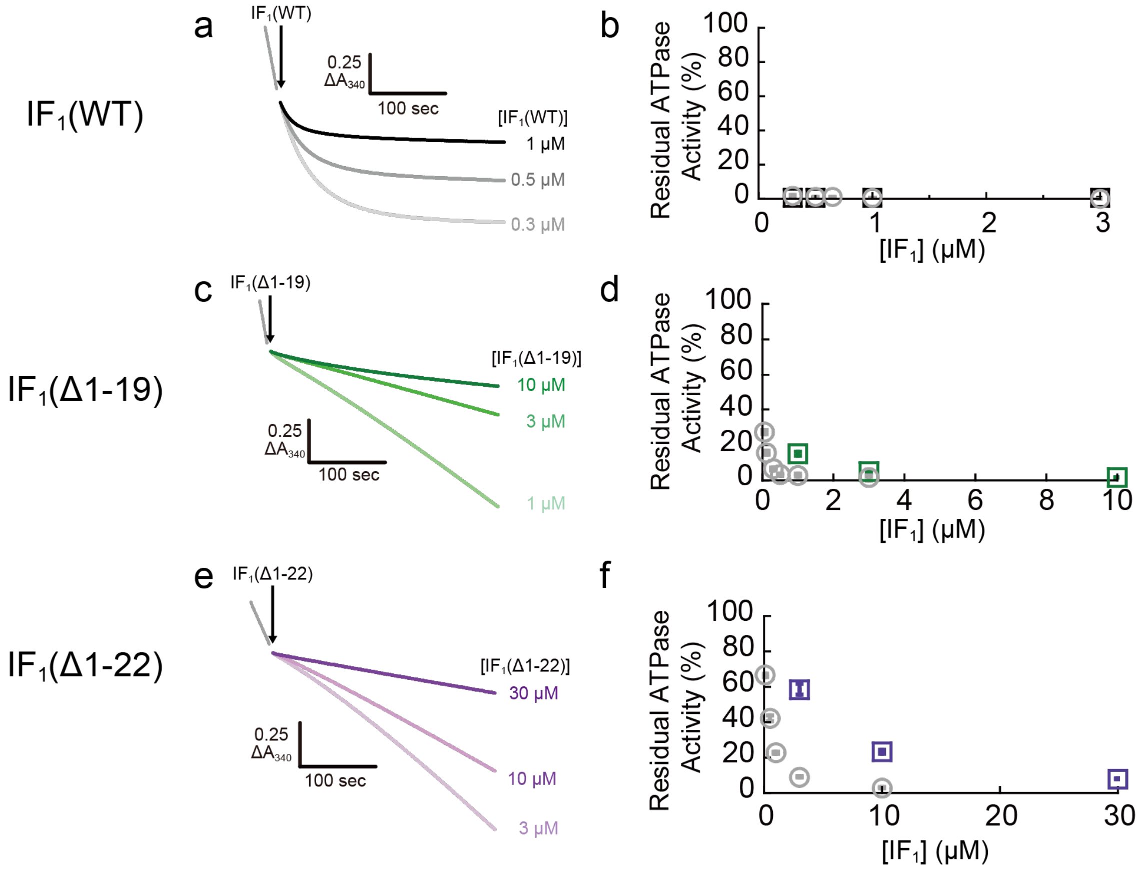

Supplementary Figure 13. Biochemical analysis under 37°C.

(a, c, e) Time courses for IF_1-inhibited *b*MF_1 at 1 mM ATP and the indicated concentrations of (a) IF_1(WT) (black), (c) IF_1(Δ1-19) (green), (e) IF_1(Δ1-22) (purple). The final concentration of *b*MF_1 was 10 nM in (a) and (c), 5 nM in (e). (b, d, f) Residual ATPase activity versus [IF_1] at the end of the measurement (350 s after IF_1 injection). Colored squares (black, green, purple) represent results at 37°C and gray circles represent results at 25°C (Supplementary Fig. 8), respectively. Symbols and error bars represent the mean value and SD, respectively ($N = 3$ for each measurement). Source data are provided in the Source Data file.

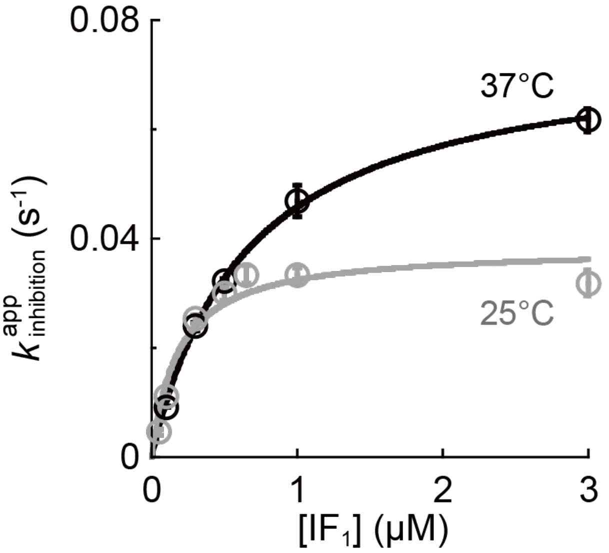

Supplementary Figure 14. $k^{app}_{inhibition}$ **plotted against [IF$_1$] at 37°C.**

$k^{app}_{inhibition}$ and $K^{IF_1}_{M}$ at 37°C were determined to be 0.076 ± 0.002 s^{-1} and 656 ± 38 nM (fitting parameter ± fitting error), respectively. Solid lines represent the fitting curve of Eq. (2). Data fitting was conducted from 2 s after IF$_1$ injection. Circles and error bars represent the mean value and SD, respectively (N = 3 for each measurement). Data at 25°C were also plotted for comparison. Source data are provided in the Source Data file.

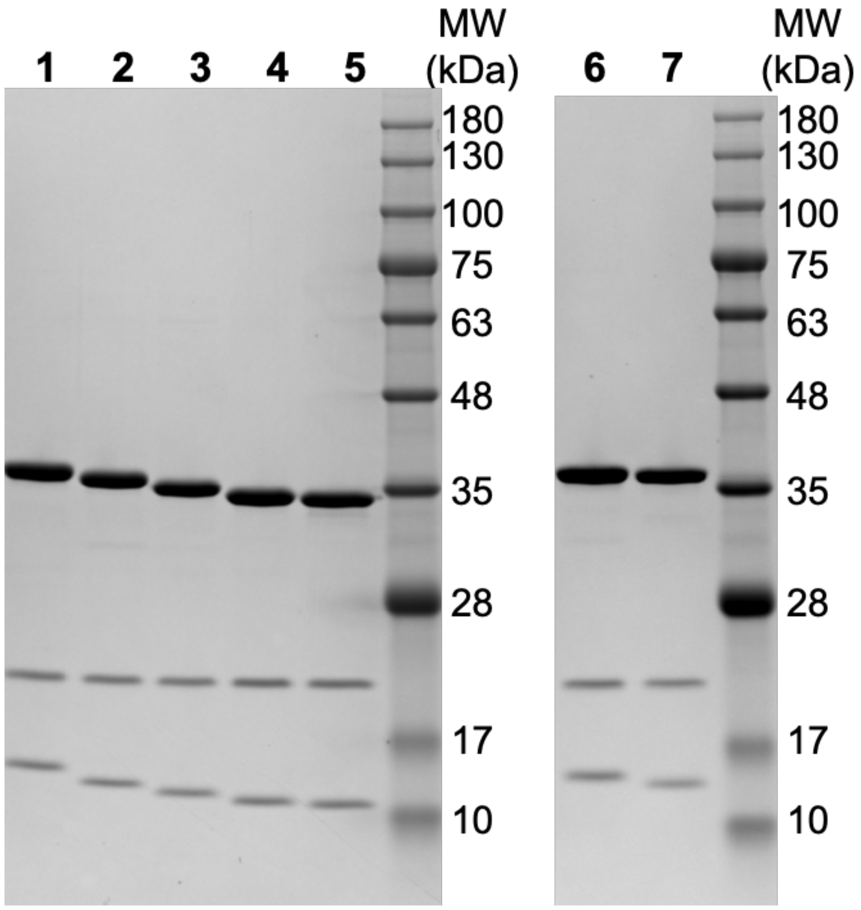

Supplementary Figure 15. SDS-PAGE analysis of (left) the N-terminal truncation IF_1s and (right) the C-terminal Alanine-substitution mutant.

Numbers indicate the bands for IF_1(WT) (lane 1, 6), IF_1(Δ1-7) (lane 2), IF_1(Δ1-12) (lane 3), IF_1(Δ1-19) (lane 4), IF_1(Δ1-22) (lane 5), and IF_1(C5 Mutant) (lane 7). A 10-20% gradient polyacrylamide gel was used for this assay. The bands around 35 kDa are the corresponding IF_1 proteins. The two lower fragmented bands (~20 kDa and ~15 kDa) should be generated by heat treatment prior to gel electrophoresis. This may be due to cleavage of red fluorescent proteins near the chromophore upon heating, as previously reported [13]. This experiment was done twice. The unprocessed gel images are provided in the Source Data file.

Supplementary Table 1. Molecular mass of IF_1 mutants.

Masses were measured by MALDI-TOF/TOF mass spectrometry and calculated from protein sequences.

IF_1	Mass (kDa)		Difference
	MALDI-TOF/TOF	Sequence	
IF_1 (Δ1-7)	32.9	32.9	0.0
IF_1 (Δ1-12)	32.3	32.3	0.0
IF_1 (Δ1-19)	31.6	31.7	+ 0.1
IF_1 (Δ1-22)	31.4	31.4	0.0
IF_1 (C5 Mutant)	33.0	33.2	+ 0.2

Supplementary Table 2. Kinetic parameters of IF_1 mutants determined by this research.

k_{lock} and $K_M^{IF_1}$ were determined from fitting of the plots of $k_{inhibition}^{app}$ versus [IF_1]. Values represent fitting parameters ± fitting errors, respectively. Experiments were performed at 1 mM ATP. Data fitting of time courses in 340 nm NADH absorbance was conducted from 2 s after IF_1 injection. N.D. means Not Determined.

IF_1	k_{lock} (s^{-1})	$K_M^{IF_1}$ (nM)
IF_1(WT)	0.034 ± 0.002	173 ± 47
IF_1(Δ1-7)	0.033 ± 0.001	111 ± 12
IF_1(Δ1-12)	0.031 ± 0.001	116 ± 22
IF_1(Δ1-19)	N.D.	N.D.
IF_1(Δ1-22)	N.D.	N.D.
IF_1(C5 Mutant)	0.032 ± 0.001	384 ± 37

Supplementary Table 3. Comparison of pause positions by inhibitors.

Experiments were performed with magnetic beads at 100 nM ATP and indicated concentrations of inhibitors. The angular distance (Δθ) was defined from the left-side ATP binding waiting dwell (Fig. 2c and Supplementary Fig. 4). Values represent mean ± SD estimated from Gaussian fitting of the plots.

Inhibitor	Concentration	Δθ (°)	Ref
AMP-PNP	500 nM	76 ± 13	Ref [14]
IF_1	5 μM	89 ± 14	Fig. 2c
NaN_3	50 mM	80 ± 8	Supplementary Fig. 4

Supplementary Table 4. Comparison of active and inactive states in the ADP inhibition observed in single-molecule rotation assay.

τ_{lp} and τ_{rot} were determined in Supplementary Fig. 1. Although histograms for pause time give two different time constants, τ_{sp} and τ_{lp}, τ_{lp} is a dominant for ADP inhibition as shown in the previous paper [10].

Experimental condition	τ_{lp} Pause time (s)	τ_{rot} Rotation time (s)	$\tau_{rot}/(\tau_{lp}+\tau_{rot})$ Active ratio (%)
ATP 1 mM	17.2	54.0	75.8
ATP 100 μM<br>ADP 100 μM<br>P_i 1 mM	31.2	70.1	69.2
ATP 100 μM<br>ADP 100 μM	28.4	26.2	48.0
ATP 100 μM<br>P_i 1 mM	26.4	72.9	73.4

Supplementary Table 5. Comparison of active and inactive states in the IF_1 inhibition observed in single-molecule rotation assay.

Parameters were determined in Supplementary Figs. 10 and 12. For IF_1(WT), IF_1(Δ1-7), IF_1(Δ1-12) and IF_1(C5 Mutant), precise pause duration times and relative active ratios were not determined because inhibited *b*MF_1 did not resume rotations after it lapsed into IF_1 inhibition. In this table, pause time for these mutants were defined as > 480 s, and approximated values for relative active ratios were given. Experiments were conducted under 100 μM ATP, 100 μM ADP and 1 mM P_i with 3 μM IF_1, except for IF_1(C5 Mutant), 30 μM.

IF_1	$\tau_{pause}^{IF_1}$ or $\tau_{lp}^{IF_1}$ Pause time (s)	$\tau_{rot}^{IF_1}$ Rotation time (s)	$\tau_{rot}^{IF_1}/(\tau_{pause}^{IF_1}+\tau_{rot}^{IF_1})$ Active ratio (%)
IF_1(WT)	> 480	15.2	< 3
IF_1(Δ1-7)	> 480	3.4	< 1
IF_1(Δ1-12)	> 480	4.1	< 1
IF_1(Δ1-19)	254	1.5	0.6
IF_1(Δ1-22)	56	2.1	3.6
IF_1(C5 Mutant)	> 480	10.3	< 2

Supplementary Table 6. Statistics of single-molecule experiments

In rotation assay, we define rotating particles as consecutive revolutions in two minutes without obvious backward step (over 120°). In manipulation experiments, data on molecules were omitted when molecules showed following behaviors after manipulation; significantly greater rotational diffusion (over 120°), attachment onto a coverslip, or dissociation from a coverslip.

Rotation assay	
Bound beads per one view (35×50 μm^2)	15~20
Ratio of rotating beads to all beads bound on the glass surface	0.5~1%

Manipulation experiment	
Total number of molecules which were subject to manipulation	19
Number of molecules which were rejected from analysis	2 (11%)

Supplementary References

1. Okazaki, K. & Hummer, G. Phosphate release coupled to rotary motion of F_1-ATPase. *Proceedings of the National Academy of Sciences* **110**, 16468–16473 (2013).
2. Gledhill, J. R., Montgomery, M. G., Leslie, A. G. W. & Walker, J. E. How the regulatory protein, IF_1, inhibits F_1-ATPase from bovine mitochondria. *Proceedings of the National Academy of Sciences* **104**, 15671–15676 (2007).
3. Eswar, N. *et al.* Comparative Protein Structure Modeling Using Modeller. *Curr Protoc Bioinformatics* **15**, 5.6.1-5.6.30 (2006).
4. Jorgensen, W. L., Chandrasekhar, J., Madura, J. D., Impey, R. W. & Klein, M. L. Comparison of simple potential functions for simulating liquid water. *J Chem Phys* **79**, 926–935 (1983).
5. Maier, J. A. *et al.* ff14SB: Improving the Accuracy of Protein Side Chain and Backbone Parameters from ff99SB. *J Chem Theory Comput* **11**, 3696–3713 (2015).
6. Meagher, K. L., Redman, L. T. & Carlson, H. A. Development of polyphosphate parameters for use with the AMBER force field. *J Comput Chem* **24**, 1016–1025 (2003).
7. Phillips, J. C. *et al.* Scalable molecular dynamics with NAMD. *J Comput Chem* **26**, 1781–1802 (2005).
8. Feller, S. E., Zhang, Y., Pastor, R. W. & Brooks, B. R. Constant pressure molecular dynamics simulation: The Langevin piston method. *J Chem Phys* **103**, 4613–4621 (1995).
9. Okazaki, K. & Hummer, G. Elasticity, friction, and pathway of γ-subunit rotation in F_oF_1-ATP synthase. *Proceedings of the National Academy of Sciences* **112**, 10720–10725 (2015).
10. Hirono-Hara, Y. *et al.* Pause and rotation of F_1-ATPase during catalysis. *Proceedings of the National Academy of Sciences* **98**, 13649–13654 (2001).
11. Kobayashi, R., Mori, S., Ueno, H. & Noji, H. Kinetic analysis of the inhibition mechanism of bovine mitochondrial F_1-ATPase inhibitory protein using biochemical assay. *The Journal of Biochemistry* **170**, 79–87 (2021).
12. Green, D. W. & Grover, G. J. The IF_1 inhibitor protein of the mitochondrial F_1F_0-ATPase. *Biochimica et Biophysica Acta (BBA) - Bioenergetics* **1458**, 343–355 (2000).
13. Gross, L. A., Baird, G. S., Hoffman, R. C., Baldridge, K. K. & Tsien, R. Y. The structure of the chromophore within DsRed, a red fluorescent protein from coral. *Proceedings of the National Academy of Sciences* **97**, 11990–11995 (2000).
14. Kobayashi, R., Ueno, H., Li, C.-B. & Noji, H. Rotary catalysis of bovine mitochondrial F_1-ATPase studied by single-molecule experiments. *Proceedings of the National Academy of Sciences* **117**, 1447–1456 (2020).
